# Hypoxia-Inducible Factor 2 Alpha Is Essential for Hepatic Outgrowth and Functions via the Regulation of *leg1* Transcription in the Zebrafish Embryo

**DOI:** 10.1371/journal.pone.0101980

**Published:** 2014-07-07

**Authors:** Tzung-Yi Lin, Chi-Fu Chou, Hsin-Yu Chung, Chia-Yin Chiang, Chung-Hao Li, Jen-Leih Wu, Han-Jia Lin, Tun-Wen Pai, Chin-Hwa Hu, Wen-Shyong Tzou

**Affiliations:** 1 Institute of Bioscience and Biotechnology, National Taiwan Ocean University, Keelung, Taiwan; 2 Institute of Cellular and Organismic Biology, Academia Sinica, Taipei, Taiwan; 3 Department of Computer Science and Engineering, National Taiwan Ocean University, Keelung, Taiwan; 4 Department of Life Sciences, National Taiwan Ocean University, Keelung, Taiwan; Institute of Cellular and Organismic Biology, Taiwan

## Abstract

The liver plays a vital role in metabolism, detoxification, digestion, and the maintenance of homeostasis. During development, the vertebrate embryonic liver undergoes a series of morphogenic processes known as hepatogenesis. Hepatogenesis can be separated into three interrelated processes: endoderm specification, hepatoblast differentiation, and hepatic outgrowth. Throughout this process, signaling molecules and transcription factors initiate and regulate the coordination of cell proliferation, apoptosis, differentiation, intercellular adhesion, and cell migration. Hifs are already recognized to be essential in embryonic development, but their role in hepatogenesis remains unknown. Using the zebrafish embryo as a model organism, we report that the lack of Hif2-alpha but not Hif1-alpha blocks hepatic outgrowth. While Hif2-alpha is not involved in hepatoblast specification, this transcription factor regulates hepatocyte cell proliferation during hepatic outgrowth. Furthermore, we demonstrated that the lack of Hif2-alpha can reduce the expression of liver-enriched gene 1 (*leg1*), which encodes a secretory protein essential for hepatic outgrowth. Additionally, exogenous mRNA expression of *leg1* can rescue the small liver phenotype of *hif2-alpha* morphants. We also showed that Hif2-alpha directly binds to the promoter region of *leg1* to control *leg1* expression. Interestingly, we discovered overrepresented, high-density Hif-binding sites in the potential upstream regulatory sequences of *leg1* in teleosts but not in terrestrial mammals. We concluded that *hif2-alpha* is a key factor required for hepatic outgrowth and regulates *leg1* expression in zebrafish embryos. We also proposed that the *hif2-alpha-leg1* axis in liver development may have resulted from the adaptation of teleosts to their environment.

## Introduction

In vertebrates, the liver is the largest internal organ and is responsible for metabolism, detoxification, digestion, and the maintenance of homeostasis. Understanding liver development not only helps us to understand the morphogenesis and development of other major organs but also provides a key to the delineation of liver carcinogenesis as well as mechanistic clues to the rational production of hepatocytes from stem cells.

In the embryo, the liver is derived from the endoderm of three germ layers. In mouse, hepatic specification starts in the ventral foregut endoderm at embryonic day 8.0 (e8.0). Subsequently, the liver diverticulum forms adjacent to the heart at e9.0. At e9.5, hepatic endoderm cells (hepatoblasts) in the liver diverticulum delaminate from the epithelium and invade the septum transversum mesenchyme (STM), thereby forming a liver bud. Hepatoblasts found in liver buds are bi-potential, with the parenchyma differentiating into the liver (hepatocytes) and the cells localized next to the portal veins differentiating into the bile ducts [1], [2].

In zebrafish, hepatogenesis is divided into three main stages: hepatoblast specification, hepatocyte differentiation, and hepatic outgrowth [3]–[5]. Cells in the anterior endodermal rod are specified into hepatoblasts at 22 hours post-fertilization (hpf) during the hepatoblast specification phase. Hepatoblasts settle on the left side of the anterior gut tube, and the liver bud begins to form approximately 26–28 hpf. Several marker genes, such as *ceruloplasmin* (*cp*), *transferrin* (*tfa*), and *liver fatty acid-binding protein* (*lfabp*), are expressed in the liver bud during the differentiation phase from 32 hpf onwards. The liver bud leaves the intestine at 50 hpf, and a rapid growth phase of the liver begins at 80–84 hpf [6]. Finally, the liver relocates to the right side from the left side by 5 days post-fertilization (dpf) [3].

For the spatial and temporal control of the developmental process of hepatogenesis, signaling molecules and transcription factors are conserved in mouse and zebrafish. The FGF, BMP, and Wnt pathways are involved in endoderm patterning and hepatoblast specification [7]–[11]; however, the anatomic localization of the adjacent tissues as the source of signaling molecules is not the same between mouse and zebrafish [12], [13]. Among the conserved transcription factors controlling hepatogenesis [3], *hhex* and *prox1* are both expressed in the zebrafish hepatic bud at 22 hpf, and both play essential roles during the delamination of hepatoblasts and liver budding [13]–[16]. Multiple *hnfs* are also involved in liver development and differentiation in mammals [17]–[20]. The transcription factors *sox17* [4], [21], *foxa1* [22], *foxa2* [4], [22], *foxa3* [4], [22], and *gata4–6* [23]–[25] are expressed in both the endoderm and the liver bud. Recently, several genes, including liver-enriched gene 1 (*leg1*) [26], were shown to be important in the outgrowth stage. Leg1 is a secretory protein, and the knockdown of *leg1* diminishes the liver expansion and can lead to a hypoplastic exocrine pancreas and intestine in the zebrafish embryo.

Hypoxia-inducible transcription factors (Hif1, Hif2, Hif3) are members of the PAS (Per-ARNT-Sim) family of basic helix-loop-helix transcription factors and are well known to be involved in metabolism, angiogenesis, erythropoiesis, cell proliferation, and apoptosis [27], [28]. Loss of both *hif1-αlpha* alleles in the mouse embryo has been shown to lead to a complete lack of cephalic vascularization, reduction in the number of somites, neural tube defects [29], the inhibition of neural crest migration [30], cardiovascular malformations, and marked cell death within the cephalic mesenchyme [31]. *hif2-alpha* is essential for catecholamine homeostasis [32] and neural [33] and hematopoietic development [34]. A knockout of *hif2-alpha* caused developmental defects in several organs, including the retina, heart, lung, liver, bone marrow and muscle [32], [35], [36]. *hif* has also been shown to participate in liver disease, liver regeneration, liver fibrosis, and hepatocellular carcinoma [37].

Recently, hypoxic cells were found in the mouse fetal liver (e11.0) by hypoxic probe staining [38]. Here, we hypothesized that *hif* is involved in the process of liver development in the zebrafish embryo. *hif2-alpha*-null mice do not survive due to a circulatory failure during mid-gestational embryonic development [32]. In contrast, the zebrafish embryo is an ideal model system to study liver development because liver development in zebrafish is mostly independent of cardiovascular and blood development [6]. Here, we demonstrated that *hif2-alpha* controls the hepatic outgrowth phase but not the liver specification phase in zebrafish embryos. Moreover, *hif2-alpha* regulates hepatic outgrowth directly by binding to the hypoxia response elements (HREs) located in the promoter regions upstream of the *leg1* gene. Additionally, we identified high-density clusters of HRE upstream of the *leg1* gene in teleosts but not in terrestrial mammals. Interestingly, when we mimicked hypoxic conditions by treating zebrafish embryos with CoCl_2,_ we detected up-regulated expression of the *hif1-alpha* target gene (*igfbp-1*) but not the *hif2-alpha* target genes (*leg1*, *birc5a*, *birc5b*).

## Materials and Methods

### Ethics statement

This study did not involve non-human primates. All experiments described in this research were performed in full accordance with the guidelines for animal experiments released by the College of Life Sciences, National Taiwan Ocean University. This study was approved by the Animal Ethics Committee at the National Taiwan Ocean University (Affidavit of Approval of Animal Use Protocol code 101045).

### Zebrafish maintenance

Adult zebrafish and Tg(*lfabp*:EGFP) transgenic fish [39] were maintained and bred as described previously [40]. The embryos were collected using natural mating and were cultured at 28.5°C.

### Mopholino and cRNA injection

The morpholinos of *hif-alpha*, *p53* [33] and *leg1* [18] were designed, and the concentration used were previously described. Briefly, we injected the translational morpholinos of *hif1-alpha*, 5′-CAGTGACAACTCCAGTATCCATTCC-3′ (6 ng/embryo), *hif2-alpha*, 5′-CGCTGTTCTCGCGTAATTCCCGCAG-3′ (6 ng/embryo), 5′-*hif3-alpha*, CCTTTTCGACGTAGAGTTCACCATC-3′ (12 ng/embryo), and *p53*, 5′-GCGCCATTGCTTTGCAAGAATTG-3′ (9 ng/embryo) at the one-cell stage. For capped-RNA synthesis, the full-length cDNAs of *hif2α* and *leg1* were cloned into the pT7TS vector and the pCS2^+^ vector, respectively [39]. After linearization, the plasmids were transcribed *in* *vitro* with T7/SP6 RNA polymerase and a mMESSAGE mMACHINE kit from Ambion (Austin, TX, USA, cat. no. AM1345/AM1340). For the rescue assay, 75 pg of purified *hif2-alpha* cRNA or 50 pg of *leg1* cRNA were co-injected with the *hif2-alpha* morpholino into one-cell stage embryos.

### Whole-mount immunostaining and TUNEL assay

Whole-mount immunostaining with a phospho-histone H3 (pH3) antibody was performed as previously described [41]. Tg(*lfabp*:EGFP) transgenic fish embryos were fixed with 4% PFA overnight at 4°C. The fixed embryos were treated with methanol overnight at −20°C and were washed three times with 1X PBT for five minutes. Washed samples were incubated with 3% bovine serum albumin for one hour at room temperature. Samples were incubated with primary antibodies for mouse anti-GFP (1:20 dilution, Invitrogen, Carlsbad, CA, USA, cat. no. A11120) and rabbit anti-pH3 Ser 10 (1:400 dilution, Santa Cruz Biotechnology, Inc., Dallas, Texas, USA, cat. no. sc-8656-R) overnight at 4°C. After being washed with PBT for 20 minutes, samples were further incubated with secondary antibodies of Alexa Fluor 488 conjugated anti-mouse IgG (1:600 dilution, Invitrogen, Carlsbad, CA, USA, cat. no. A11001) and Alexa Fluor 568 conjugated anti-rabbit IgG (1:600 dilution, Invitrogen, Carlsbad, CA, USA, cat. no. A11011) for two hours at room temperature. Finally, samples were washed three times with 1X PBT for ten minutes and kept at 4°C. We performed the unpaired *t-test* to compare the cell numbers between *hif-2 alpha* morphants and wild-type embryos.

TUNEL assays were performed with an *in* *situ* cell death detection kit using TMR red (Roche, cat. no. 12 156 792 910). Briefly, fixed 4 dpf Tg(*lfabp*:EGFP) transgenic fish embryos were incubated in permeabilization solution for 15 minutes on ice. The samples were labeled with the TUNEL reaction mixture for one hour at 37°C in the dark. Finally, the labeled samples were washed three times with 1X PBT for ten minutes and kept at 4°C. All images were acquired using a fluorescence microscope (OLYMPUS BX 51, Olympus, Tokyo, Japan).

### Embryonic cell isolation and fluorescence activated cell sorting (FACS)

The counting of EGFP^+^ cells in Tg(*lfabp*:EGFP) embryos was based on previously described methods [42]. The embryos were cultured at 28.5°C until 4 dpf. Every 20 embryos were collected and placed in 1 ml phosphate buffered saline (PBS) containing 0.25% trypsin and 1 mM EDTA for 20 min at 28.5°C. During the trypsin digestion, embryos were pipetted with a 200-µl tip every 10 min. The reaction was stopped by adding lamb serum to 10%. The cells were centrifuged for 3 min at 3000 rpm. After discarding the supernatant, the cell pellets were suspended in Leibovitz medium L15 containing 1% lamb serum, 0.8 mM CaCl_2_, penicillin 50 U/ml and streptomycin 0.05 mg/ml. The cell counting was analyzed by BD FACSCanto II.

### Whole-mount in situ hybridization

Antisense digoxigenin (Roche Applied Science, Indianapolis, IN, USA, cat. no. 1 277 073) probes for *hif1-alpha* (XM_005160492.1, bases 2723–3063), *hif2-alpha* (DQ375242, bases 2557–3137), *hif3-alpha* (NM_200405.1, bases 1967–2959), *lfapb* (NM_001044712, bases 29–346), *hhex* (NM_130934, bases 10–717), *prox1* (NM_131405.2, bases 179–1397), *ins* (NM_131056, bases 71–334), *try* (NM_131708.1, bases 219–645), *ifabp* (NM_131405.2, bases 15–524) and *leg1* (NM_001100056, bases 1–1095) were generated by *in* *vitro* transcription using T7 or SP6 RNA polymerase as described previously [40]. Fixed zebrafish embryos were treated with methanol overnight and were then rehydrated with 1X PBT. Rehydrated samples were treated with proteinase K (10 µg/ml) at room temperature and fixed with 4% PFA for 30 minutes to remove enzyme activity. The secondary fixed samples were incubated with a hybridization buffer (50% deionized formamide, 5X SSC, 0.1% Tween-20, 50 µg/ml heparin, 500 µg/ml RNase-free tRNA) at 65°C for three hours after rinsing with 1X PBT. The samples were hybridized with 150 ng of antisense probe in hybridization buffer overnight at 65°C. The samples were washed by changing the buffer from 2X SSC to 0.2X SSC at 65°C gradually. After rinsing with 1X PBT, the samples were incubated for three hours at room temperature in 1% blocking reagent (Roche Applied Science, Indianapolis, IN, USA, cat. no. 11 096 176 001). The samples were incubated with Anti-Digoxigenin-AP, Fab fragments (1:10000 dilution, Roche Applied Science, Indianapolis, IN, USA, cat. no. 11 093 274 910) overnight at 4°C. The samples were colored with Nitroblue tetrazolium chloride (NBT) (Roche Applied Science, Indianapolis, IN, USA, cat. no. 11 383 213 001) and 5-Bromo-4-chloro-3-indolyl-phosphate, 4-toluidine salt (BCIP, 4-toluidine salt) (Roche Applied Science, Indianapolis, IN, USA, cat. no. 11 383 221 001) in the dark after 1X PBT wash. Finally, the coloring reaction was blocked by 4% PFA incubation for 20 minutes. All images were acquired using a microscope (OLYMPUS BX 51, Olympus, Tokyo, Japan).

### Quantitative Reverse-Transcription Polymerase Chain Reaction (qPCR)

Total RNA from zebrafish embryos was purified and reverse transcribed to generate complementary DNA (cDNA). Quantitative RT-PCR was performed using a Bio-Rad, iQ5 Gradient Real Time PCR System (Bio-Rad, Hercules, CA, USA) as described previously [33]. The primer sequences are listed in Table S1 [33], [43]–[46]. The qPCR reaction was as follows: 95°C for 3 minutes, 40 cycles of 95°C for 15 seconds, and 60°C for 1 minutes. The expression levels of β-actin mRNA were used to normalize the relative mRNA abundance. Relative quantification was determined using the ΔΔCt method: relative expression = 2^−ΔΔCt^. The mean values and standard deviations were calculated using the iQ5 Optical System Software. The experiment was performed in triplicate. Data were analyzed with the unpaired *t-test* to compare the treated groups with the untreated group (wild type). *indicates that the significant difference was *p*<0.05.

### Chromatin immunoprecipitation (ChIP)

ChIP was performed using a Magna ChIP G Chromatin Immunoprecipitation Kit (Millipore, Billerica, MA, USA, cat. no. 17–611) as described previously [33]. Adult zebrafish liver tissue was cross-linked with 1% formaldehyde, and unreacted formaldehyde was quenched by 125 mM glycine. The liver tissue was homogenized by a tissue grinder and lysed in Cell Lysis Buffer (Millipore, Billerica, MA, USA, cat. no. CS200634) after being washed with 125 mM glycine. The cell pellets were suspended in Nuclear Lysis Buffer (Millipore, Billerica, MA, USA, cat. no. CS200623) after centrifugation and washed with 1X PBS. The cell lysates were sonicated on wet ice with twenty 30-s pulses (30-s pause between pulses) by a sonicator (MISONIX, Farmingdale, NY, USA, cat. no. S-4000), and the DNA was sheared to ∼200–500 base pairs in length. Recombinant protein G covalently bound to magnetic beads was incubated with a polyclonal antibody against anti-Hif2-alpha (GeneTex, Irvine, CA, USA, ChIP grade rabbit polyclonal antibody, cat. no. GTX103707) or normal Rabbit IgG (Millipore, Billerica, MA, USA, cat. no. 12–370) as the negative control at 4°C for 3 hours before the sheared DNA was incubated with the magnetic bead-linked antibody overnight at 4°C. The beads were washed once with different wash buffers (Millipore, Billerica, MA, USA, cat. no. 20–154, cat. no. 20–155, and cat. no. 20–156) for 10 minutes. All wash procedures were performed with a magnetic separator (Millipore, Billerica, MA, USA, Magna Grip Rack (8 Well), cat. no. 20–400). The washed immunocomplexes were eluted with TE buffer, and the protein–DNA complexes were reverse crosslinked by proteinase K digestion at 62°C for 2 hours followed by 95°C for 10 minutes. Finally, DNA was purified with spin columns and eluted using elution buffer. For PCR amplification, immunoprecipitated DNA and input DNA (unimmunoprecipitated) were used as templates. The PCR primers used in the ChIP assay are listed in Table S2.

### CoCl_2_ treatment of zebrafish embryos

The coding sequences of zebrafish *hif1-alpha* and zebrafish *hif2-alpha* were subcloned into pCMV-Tag 2A (Agilent Technologies, SC, California, USA, cat. no. 211172). The templates were generated by PCR, and we synthesized capped mRNA of FLAG-tagged *hif-1 alpha* and FLAG-tagged *hif-2 alpha* with T3 RNA polymerase and an mMESSAGE mMACHINE kit from Ambion (Austin, TX, USA, cat. no. AM1348). The zebrafish embryos were injected with 2400 pg mRNA of FLAG-tagged *hif-1 alpha* and 2400 pg mRNA of FLAG-tagged *hif-2 alpha* for CoCl_2_ treatment, respectively. The un-injected and injected zebrafish embryos were treated with 10 mM CoCl_2_ in Petri dishes at 28.5°C from 48 hpf to 72 hpf [47]. *igfbp-1* had been reported to be up-regulated following CoCl_2_ treatment [47], [48] and was therefore used as the positive control in the qPCR experiments.

### Western blot

The total proteins of CoCl_2_-treated zebrafish embryos were extracted with SDS lysis buffer (0.05% SDS, 200 mM Tris Base, 5 mM EDTA, 6 M Urea) and were then separated by 8% SDS-PAGE. We transferred the proteins to Immun-Blot PVDF Membranes (Bio-Rad, Hercules, CA, USA, cat. no. 162–0177) at 100 volts for 2 hours. The PVDFs were blocked with 3% milk in TBST (TBS containing 0.1% Tween-20) for 1 hour at room temperature. After blocking, the PVDFs were incubated with rabbit anti-FLAG Tag antibody (1:2000 dilution, Cell Signaling, Danvers, MA, USA, DYKDDDDK Tag Antibody, cat. no. 2368S) or mouse anti-tubulin antibody (1:2000 dilution, GeneTex, Irvine, CA, USA, cat. no. GTX628802) overnight at 4°C. The PVDFs were washed with TBST and were incubated with Goat Anti-mouse IgG-HRP (1:50000 dilution, Jackson ImmunoResearch Laboratories, Inc., West Grove, PA, USA, cat. no. 115-001-003) or Donkey anti-rabbit IgG-HRP (1:10000 dilution, GE Healthcare Life Sciences, cat. no. NA934). After washing with TBST, the membranes were visualized by WesternBright ECL Chemiluminescent HRP Substrate (advansta, Menlo Park, California, USA, cat. no. K-12045-D50). The intensity of bands on the western blots was measured by ImageJ software.

### HRE cluster identification

The sequences upstream of the *leg1* gene in terrestrial mammals (Alpaca, Bushbaby, Cat, Chimpanzee, Cow, Dog, Elephant, Ferret, Gorilla, Guinea pig, Hedgehog, Human, Horse, Hyrax, Kangaroo rat, Rhesus macaque, Marmoset, Megabat, Mouse lemur, Mouse, Opossum, Orangutan, Panda, Pig, Pika, Platypus, Rabbit, Rat, Rock hyrax, Shrew, Sloth, Squirrel, Tarsier, and Tasmanian devil), birds (Chicken, Duck, Flycatcher, Turkey, and Zebra finch), reptiles (American alligator, Chinese softshell turtle, and Lizard), and teleosts (Cod, Zebrafish, Fugu, Medaka, Platyfish, Stickleback, and Tilapia) were downloaded from the UCSC genome database (http://genome.ucsc.edu/) and the Ensembl genome database (http://www.ensembl.org/index.html). We chose a region 10 kbps upstream of the translation start site (without including the +1) of the *leg1* gene for HRE mapping. We calculated the inter-HRE distances between each HRE and its first neighbor, together with the distance between each HRE and its second neighbor. We used a greedy algorithm to identify high-density HRE clusters. The greedy algorithm scans genomic sequences using a window size of 500 bps and identifies the cluster starting from the window containing the highest number of HREs in a recursive fashion. We performed Fisher's exact test to assess if there is a significant difference between the number of HREs from the terrestrial mammals and the teleosts.

## Results

### *Hif2-alpha* is expressed in the liver in zebrafish embryos

**Figure 1:**
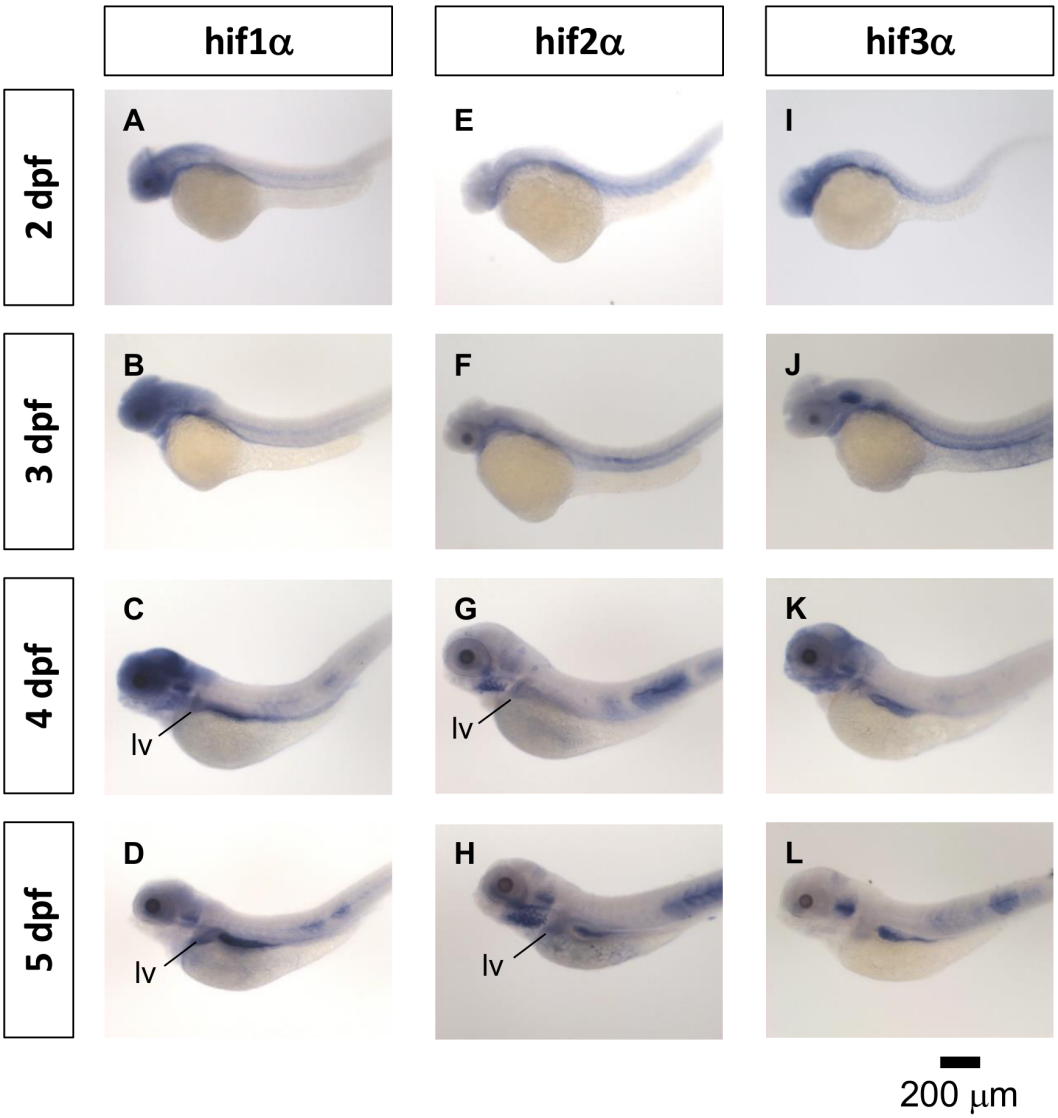
Gene expression pattern of *hif-1alpha, hif-2 alpha* and *hif-3 alpha* in zebrafish embryos. The expression patterns of *hif1-alpha* (A–D), *hif2-alpha* (E–H) and *hif3-alpha* (I–L) were performed with anti-sense probes by WISH in zebrafish embryos at 2–5 dpf. WISH, whole-mount in situ hybridization. dpf, days post-fertilization. lv, liver.

We analyzed the gene expression pattern of *hif1-alpha*, *hif2-alpha*, and *hif3-alpha* by whole-mount in situ hybridization (WISH). The results revealed that *hif1-alpha* was expressed in the head, notochord and intestine at 2, 3, 4 and 5 dpf and was expressed in the liver at 4 and 5 dpf (Fig. 1A–D). *Hif2-alpha* was expressed in the notochord and somites at 2 and 3 dpf and expressed in the pharyngeal arches, liver and intestine at 4 and 5 dpf (Fig. 1E–H). *Hif3-alpha* was expressed in the head and inner ear at 2, 3, 4 and 5 dpf and was expressed in the intestine at 4 and 5 dpf (Fig. 1I–L; we also performed WISH using the sense probes of *hif1-alpha*, *hif2-alpha*, and *hif3-alpha*, as shown in Fig. S1).

### Knockdown of *hif2-alpha* confers a small liver phenotype

**Figure 2:**
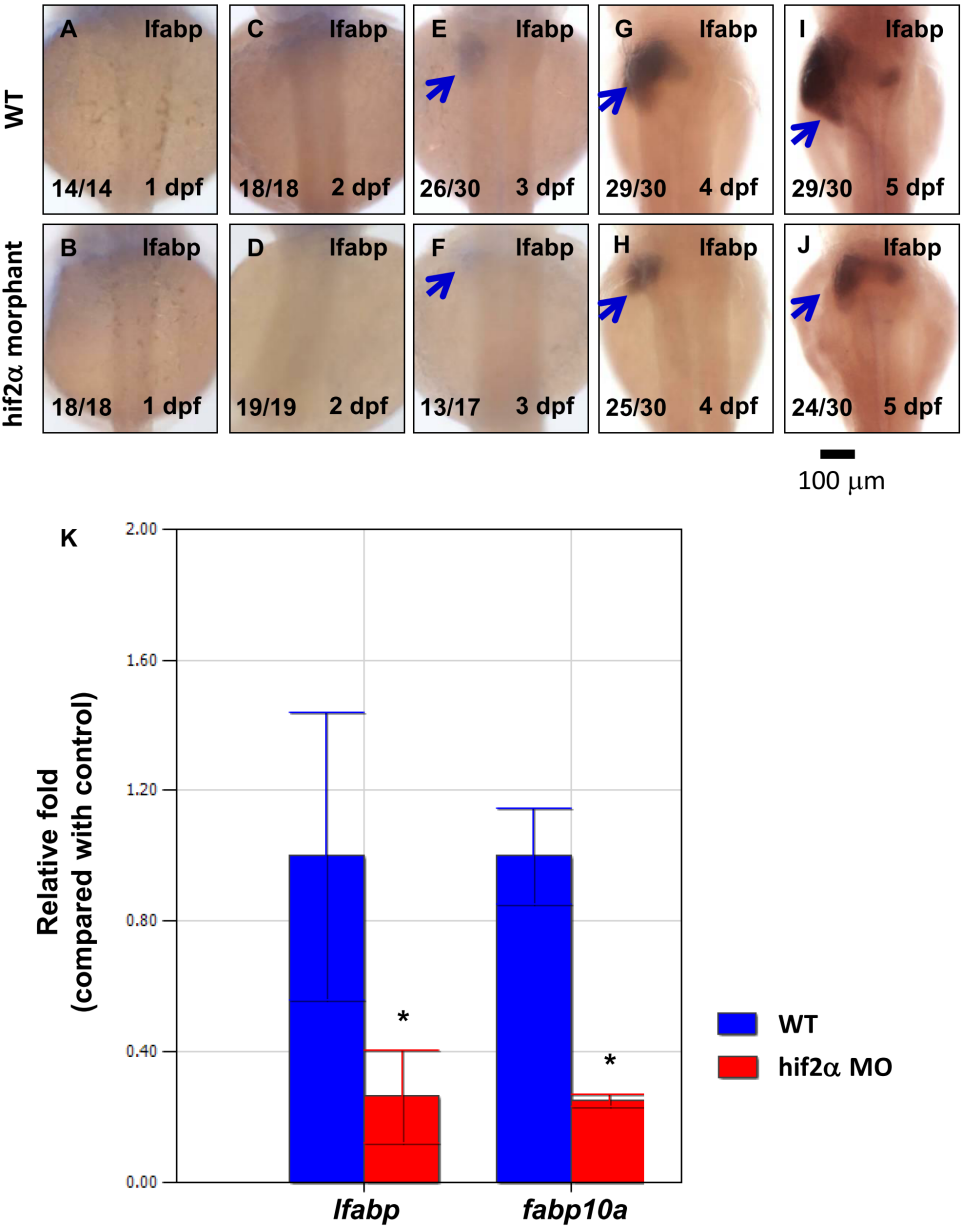
Knockdown of *hif2-alpha* confers a small liver phenotype. (A–J) Development of the embryonic liver was monitored by the expression pattern of the *lfabp* gene in wild-type embryos and *hif2-alpha* ATG-MO-injected embryos at 1–5 dpf by WISH, thereby revealing a small liver phenotype in *hif2-alpha* morphants. (K) Expression of the *lfabp* and *fabp10a* genes was investigated in wild-type embryos and *hif2-alpha* morphants at 5 dpf by qPCR (n = 30). Expression was normalized to *β*-actin. WT, wild type. The experiment was performed in triplicate, error bars indicate S.D. *, *p*<0.05, unpaired *t-test*.

Our initial goal was to understand the role *hif2-alpha* plays during liver development. First, we used the loss-of-function approach by injecting a morpholino molecule with a sequence matching the translation initiation site around *hif2-alpha* (*hif2-alpha* ATG-MO). We found that the size of the liver was reduced in *hif2-alpha* morphants compared to wild-type embryos by assessing the hepatocyte marker gene *lfabp* starting from 3 dpf (Fig. 2A–2J). To quantify the effect on the liver by *hif2-alpha*, we assessed *lfabp* expression levels in zebrafish embryos on 5 dpf by qPCR. *lfabp* expression levels revealed a 70% down-regulation in *hif2-alpha* morphants compared with wild-type embryos (Fig. 2K). These results suggest that *hif2-alpha* is required for the embryonic development of the zebrafish liver.

### *Hif2-alpha* is a major member of the hif family involved in liver development

**Figure 3:**
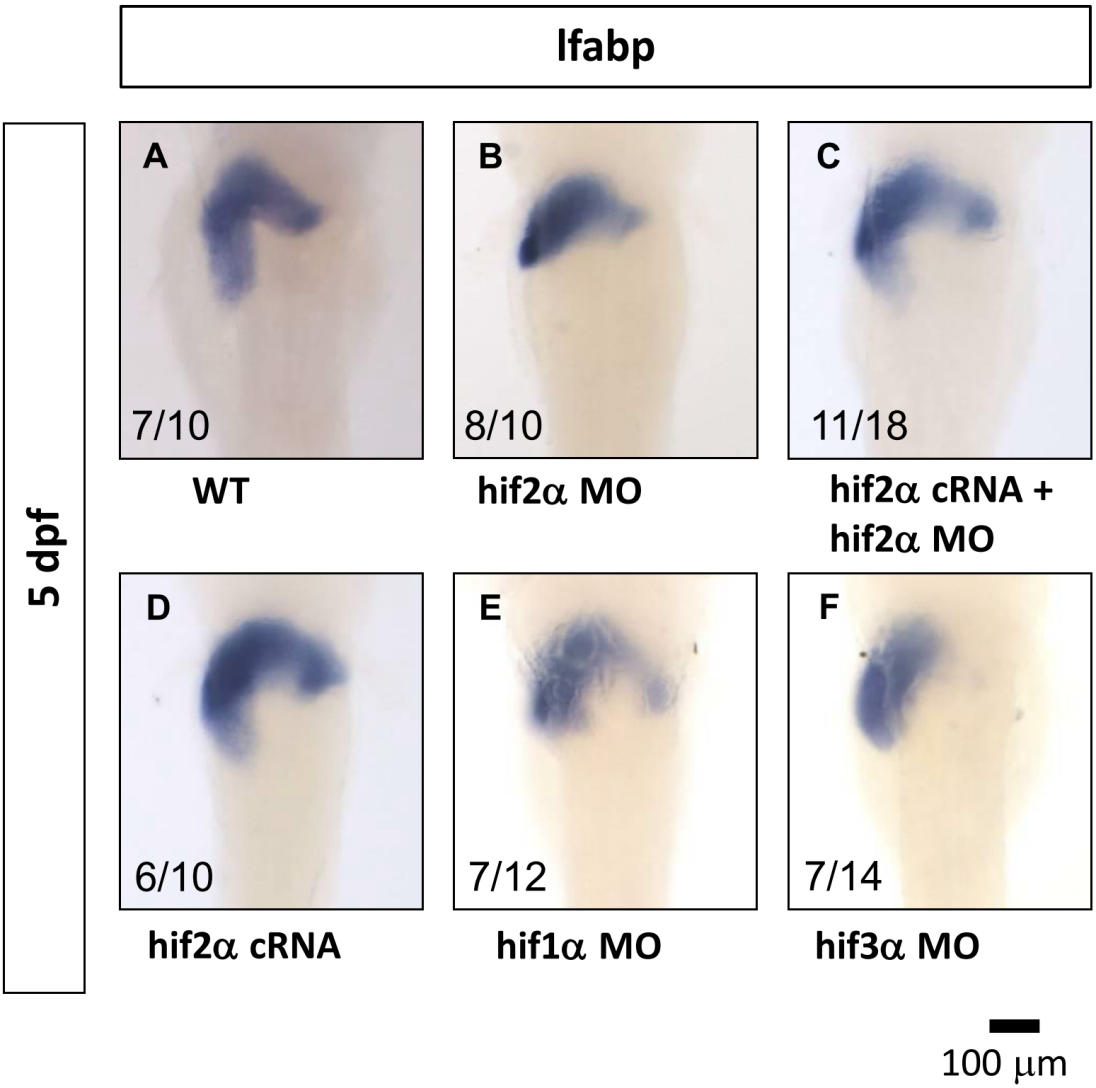
*Hif2-alpha* plays a major role in liver development. The specificity of knockdown by MO was demonstrated by monitoring *lfabp* gene expression at 5 pdf by WISH in wild-type embryos (A) and embryos injected with *hif2-alpha* ATG-MO (B). The small liver phenotype resulting from the knockdown of *hif2-alpha* can be rescued by co-injection of *hif2-alpha* mRNA (C). ATG-MO of *hif2-alpha* but not *hif1-alpha* caused the small liver phenotype (E). ATG-MO of *hif3-alpha* also caused a slight effect on liver development (F).

In the previous section, we proved that *hif2-alph*a morphants result in a small liver phenotype (Fig. 2). Here, we performed the rescue experiment to assess the specificity of the *hif2a-alpha* ATG-morpholino. The small liver phenotype resulting from the knockdown of *hif2-alpha* (Fig. 3B) can be rescued by co-injection with *hif2-alpha* mRNA (Fig. 3C). The *hif2-alpha* over-expressing zebrafish embryos revealed no obvious side effects (Fig. 3D) compared to WT embryos (Fig. 3A). Furthermore, we employed morpholino molecules specific to *hif1-alpha* and *hif3-alpha*, respectively, to examine if these two members of the *hif* family are also required for liver development. Knockdown of *hif1-alpha* does not have any conspicuous effect on liver development (Fig. 3E). However, the knockdown of *hif3-alpha* caused a slight effect on liver development (Fig. 3F). Additionally, to examine whether the *hif2-alpha* morpholino-induced effect on liver development in zebrafish embryos is mediated by *p53* activity, we concurrently knocked down *p53* and *hif2-alpha* using translational morpholinos. This result demonstrates that the depletion of p53 expression did not change the effect of the *hif2-alpha* morpholino on hepatic outgrowth (data no shown).

The small liver phenotype could result from a side effect of the *hif2-alpha* ATG-MO. When we concurrently injected *hif2-alpha* ATG-MO and *hif2-alpha* mRNA (which was not recognized by the *hif2-alpha* morpholino), the small liver phenotype was completely rescued by the ectopic *hif2-alpha* expression, as demonstrated by *in* *situ* hybridization (Fig. 3E). This result suggests that the *hif2-alpha* ATG-MO has minimal off target effects.

### *Hif2-alpha* is required for hepatic outgrowth but not specification

**Figure 4:**
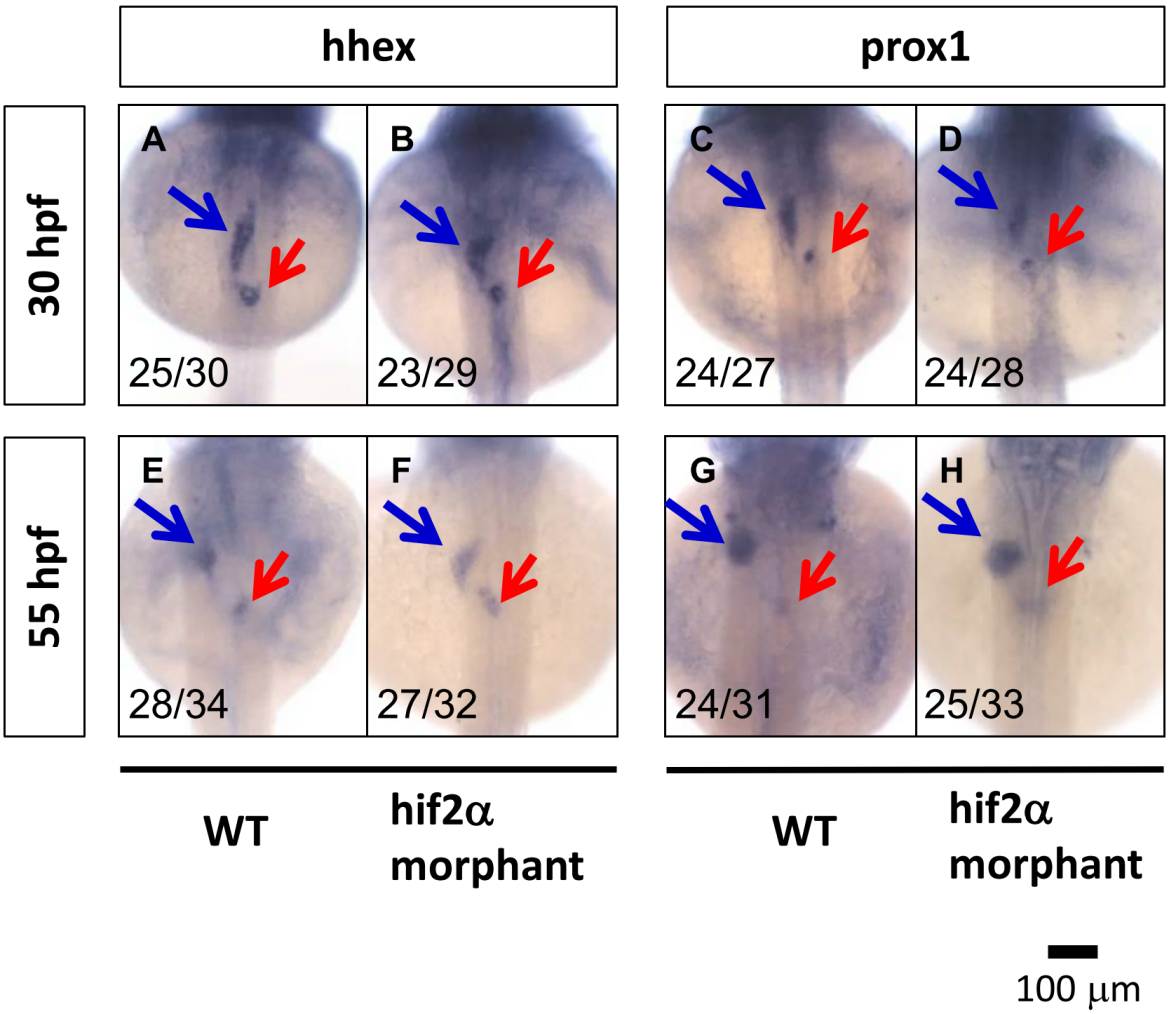
*Hif2-alpha* is not required for liver specification in zebrafish embryos. Liver specification in *hif2-alpha* morphants was detected through the expression of the *hhex* and *prox1* genes. The expression of embryonic liver specification genes, *hhex* (A, B, E, F) and *prox1* (C, D, G, H), were examined at 30 hpf (A–D) and 55 hpf (E–H) in wild-type and *hif2-alpha* ATG-MO-injected embryos by WISH.

To further examine if the knockdown of *hif2-alpha* has any effects on hepatoblast specification, we investigated the expression pattern of the *hhex* and *prox1* genes. At 30 hpf (beginning of budding) and 55 hpf (completion of budding), we found that the gene expression of *hhex* and *prox1* in *hif2-alpha* morphants is not affected compared with wild-type embryos (Fig. 4). These results demonstrate that *hif2-alpha* is required during the hepatic outgrowth phase but not in the liver specification phase in zebrafish embryos.

### Knockdown of *hif2-alpha* impairs liver cell proliferation but does not increase cell apoptosis

**Figure 5:**
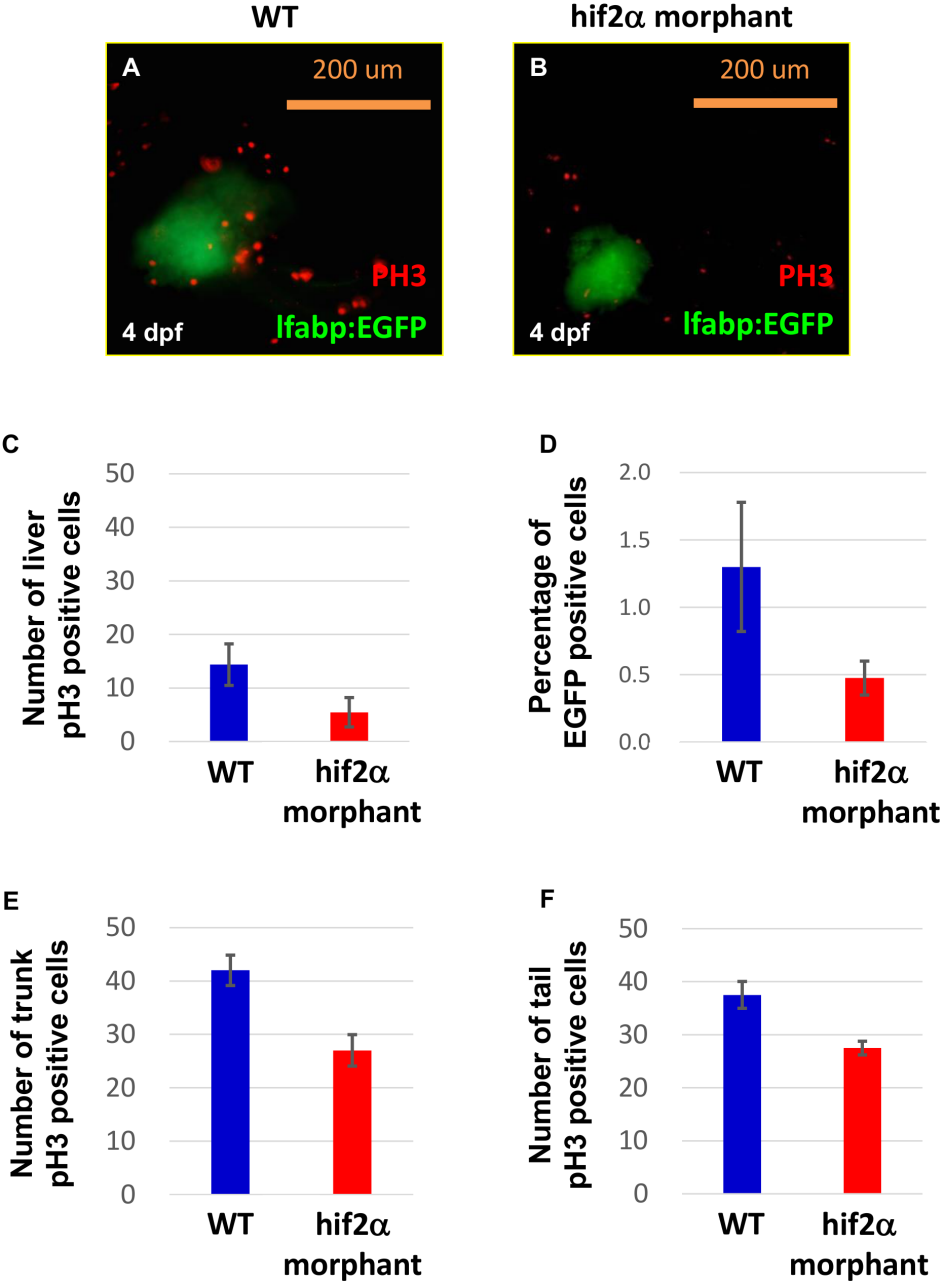
Knockdown of *hif2-alpha* damages liver cell proliferation. WT (Tg(*lfabp*:EGFP)) embryos (A) and *hif2-alpha* ATG-MO-injected embryos (B) were examined for liver cell proliferation using an anti-pH3 antibody at 4 dpf. Cell proliferation in *hif2-alpha* ATG-MO-injected embryos was reduced compared with WT embryos. (C) Quantification of pH3-positive cells in the liver (n = 11, *p*<0.05). (D) The EGFP-positive cells were counted by FACS. Quantification of pH3-positive cells in the trunk (E) and tail (F) (n = 4, *p*<0.05).

The small liver phenotype in *hif2-alpha* morphants could result from decreased cell proliferation and/or cell apoptosis. We detected less proliferation (an average decrease of 62%) in the hepatocytes of *hif2-alpha* morphants compared to wild-type embryos using pH3 as the proliferation marker at 4 dpf (Fig. 5C, n = 11, *p*<0.05). Less proliferation in the liver of *hif2-alpha* morphants could result in cell number changes. Therefore, we assayed the number of hepatocytes labeled with EGFP in Tg(*lfabp*:EGFP) by FACS. The results revealed that the EGFP-positive cells were reduced by approximately 63% on average in *hif2-alpha* morphants compared to wild-type embryos (Fig. 5D, n = 4, p<0.05). We also detected proliferation in other tissues. The number of proliferating cells was lower in the trunk (an average decrease of 35%) and the tail (an average decrease of 27%) in *hif2-alpha* morphants compared to wild-type embryos (Fig. 5E–F, n = 4, *p*<0.05 and Figure S2). However, TUNEL assays did not show an increase of cell apoptosis at 4 dpf in the hepatocytes of *hif2-alpha* morphants compared to wild-type embryos (Figure S3, n = 5). We concluded that *hif2-alpha* reduces cell proliferation in the liver of zebrafish embryos.

### *Hif2-alpha* is required for the expansion of exocrine pancreas and intestine

**Figure 6:**
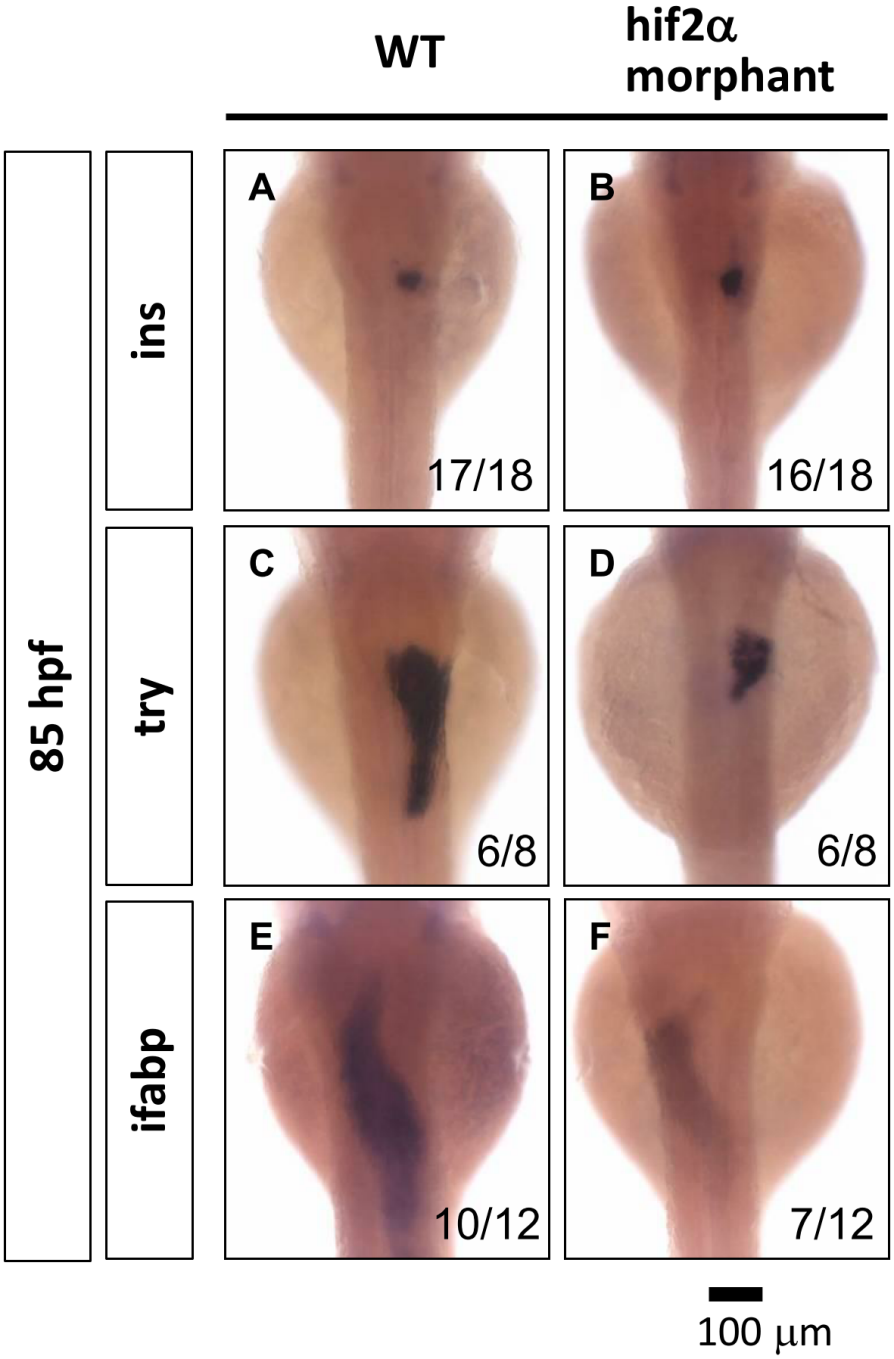
*Hif2-alpha* is required for the expansion of the exocrine pancreas and the intestine. The development of the embryonic pancreas and the intestine was monitored by examining the gene expression pattern of the endocrine pancreas (*ins*) (A, B), the exocrine pancreas (*try*) (C, D), and the intestine (*ifabp*) (E, F) in wild-type embryos compared with *hif2-alpha* ATG-MO-injected embryos at 85 hpf by WISH.

The liver, pancreas and intestine are all derived from the endoderm. We explored the effects of the depletion of *hif2-alpha* on the development of the pancreas and the intestine. In the pancreas, we assessed the marker gene *insulin (ins)* for the endocrine pancreas and *trypsin (try)* for the exocrine pancreas. We found that the development of the endocrine pancreas is not affected in *hif2-alpha* morphants (Fig. 6A, 6B), but we detected a smaller exocrine pancreas in *hif2-alpha* morphants compared to wild-type embryos at 85 hpf (Fig. 6C, 6D). In the intestine, the gene expression of the intestine marker gene intestinal fatty acid binding protein *(ifabp)* is significantly decreased in *hif2-alpha* morphants (Fig. 6E, 6F). These results suggest that *hif2-alpha* is involved in the organogenesis of the exocrine pancreas and the intestine.

### *Hif2-alpha* knockdown affects lipid metabolism but not EPO production in zebrafish embryos

**Figure 7:**
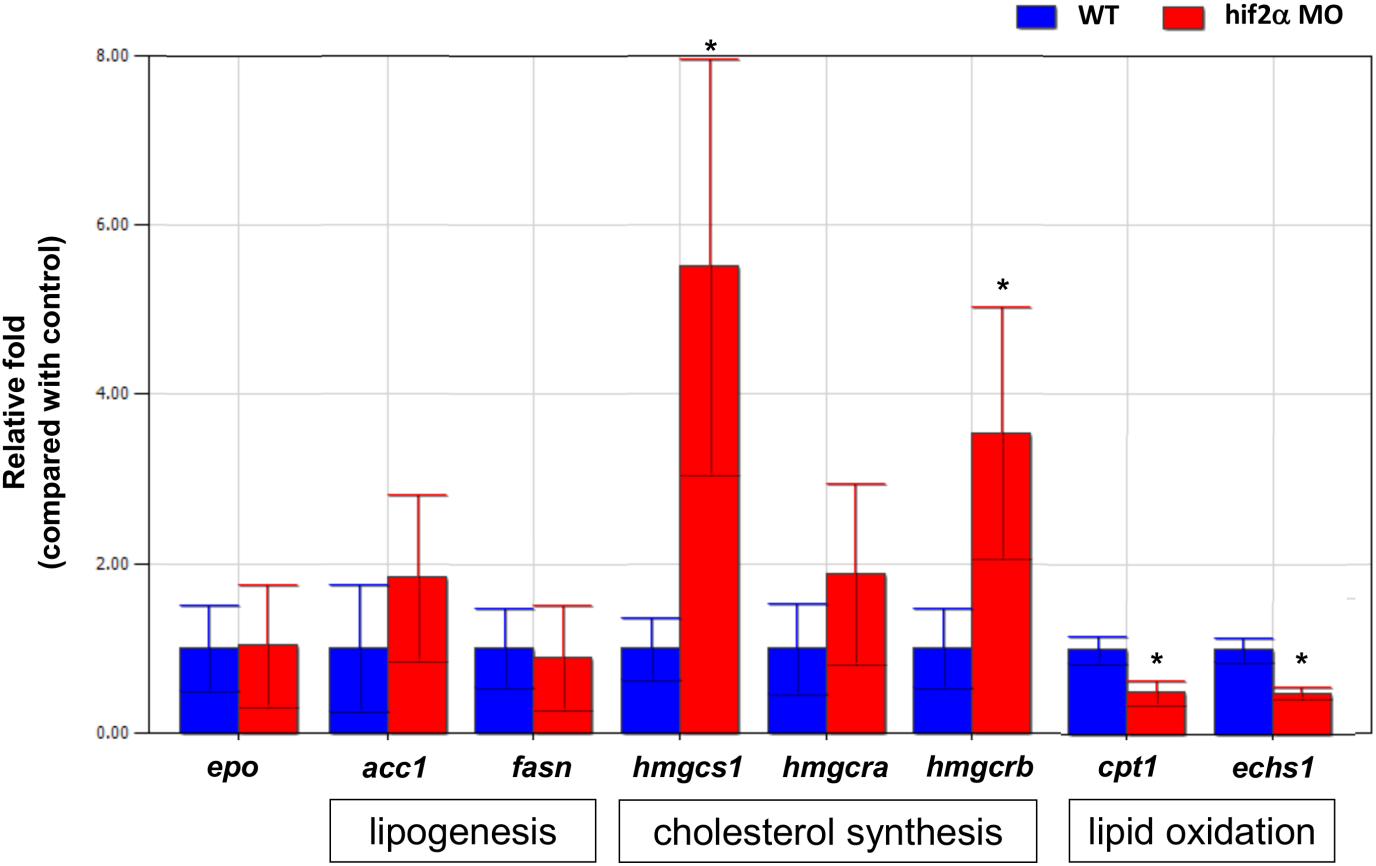
*Hif2-alpha* knockdown affects lipid metabolism but not EPO production in zebrafish embryos. The expression of EPO and the genes involved in lipid metabolism were investigated in wild-type embryos and *hif2-alpha* morphants at 5 dpf by qPCR (n = 30). Expression was normalized to *β*-actin. WT, wild type. The experiment was performed in triplicate, error bars indicate S.D. *, *p*<0.05, unpaired *t-test*.

In mammals, Hif2-alpha had been shown to affect both EPO production [49] and lipid metabolism [50]. To investigate if *hif2-alpha* knockdown affects EPO production and lipid metabolism in zebrafish embryos, we checked the gene expression of *acc1*, *fasn*, *hmgcs1*, *hmgcra*, *hmgcrb*, *cpt1* and *echs1* for lipid metabolism and *epo* for *epo* production. We found that the gene expression of *hmgcs1* and *hmgcrb* (cholesterol synthesis) increased and the *cpt1* and *echs1* (lipid oxidation) decreased significantly in *hif2-alpha* morphants compared to wild-type embryos at 5 dpf. However, we did not find any significant changes of the gene expression of *epo*, *hmgcra* (cholesterol synthesis), *acc1* and *fasn* (lipogenesis) in *hif2-alpha* morphants compared to wild-type embryos at 5 dpf (Fig. 7). These results indicated that *hif2-alpha* affects lipid metabolism (cholesterol synthesis in particular) in zebrafish embryos but not EPO production.

### *Leg1* functions downstream of *Hif2-alpha* to control hepatic outgrowth

We demonstrated that the depletion of *hif2-alpha* significantly decreased the size of the liver, exocrine pancreas, and intestine but not the endocrine pancreas. Zebrafish embryos injected with a morpholino against *liver-enriched gene 1* (*leg1*) mRNA have a similar phenotype to *hif2-alpha morphants*; a depletion of *leg1* also decreased the size of the liver, exocrine pancreas and intestine but not the endocrine pancreas [26]. The hepatoblast markers were not affected in *leg1* morpahants or *hif2-alpha* morphants (Fig. 4 and Fig. S4) [26]. Therefore, we hypothesized that *hif2-alpha* and *leg1* function in the same pathway.

**Figure 8:**
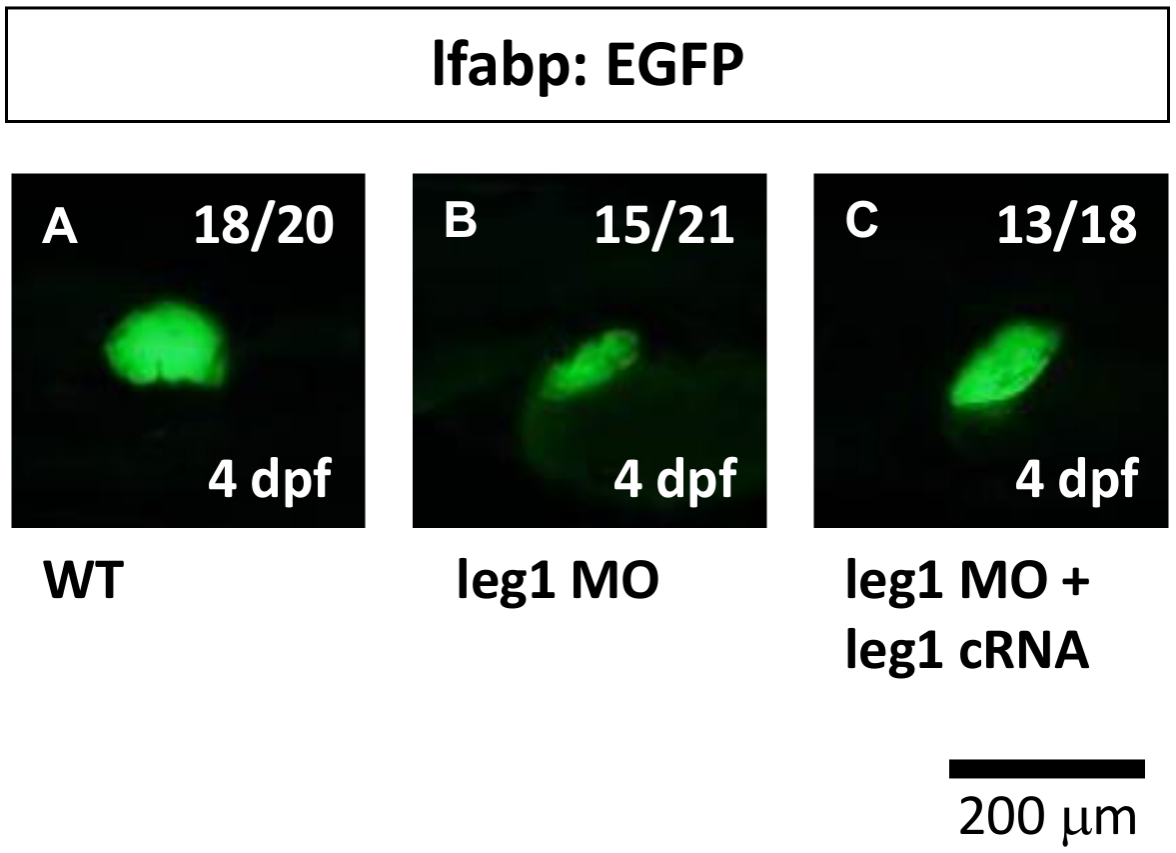
*leg1* is required for hepatic outgrowth in zebrafish embryos. The expression pattern of the *lfabp* gene was examined in Tg(*lfabp*:EGFP) embryos (A) and compared with *leg1* ATG-MO-injected embryos (0.5 µM) (B) as well as embryos co-injected with *leg1* ATG-MO (0.5 µM) and *leg1* cRNA (C).

Initially, we investigated the effect of the knockdown of *leg1* on hepatic outgrowth. We found that the size of the liver was significantly reduced in *leg1* morphants of Tg(*lfabp*:EGFP) at 4 dpf. We also conducted a rescue experiment by co-injecting *leg1* morphants with *leg1a* mRNA in Tg(*lfabp*:EGFP) transgenic fish. While *leg1* morphants presented a small liver phenotype, we found that *leg1* morphants rescued by *leg1a* mRNA presented a significantly restored liver size at 4 dpf, demonstrating the specificity of the *leg1* morpholino (Fig. 8).

**Figure 9:**
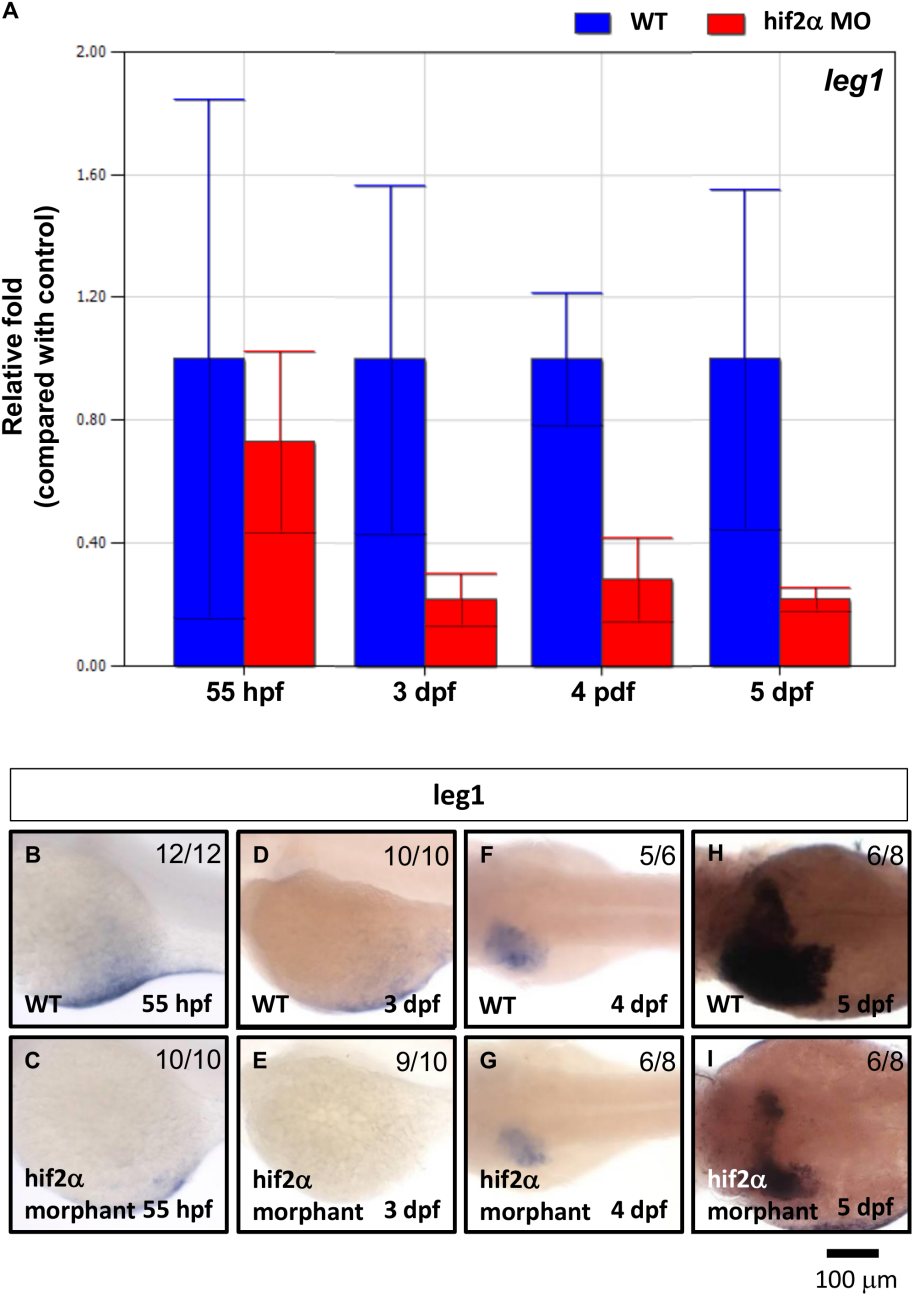
*Leg1* expression was down-regulated in *hif2-alpha* morphants. *leg1* gene expression was examined in wild-type embryos and compared with *hif2-alpha* ATG-MO-injected embryos at 3–5 dpf by qPCR (A) and WISH (B). Expression was normalized to *β-actin*.

Next, we found that approximately 70% of *leg1* gene expression was abolished in *hif2-alpha* morphants compared with wild-type embryos at 3–5 dpf but not at 55 hpf (Fig. 9). This result indicates that *leg1* functions downstream of *hif2-alpha* to control hepatic outgrowth.

**Figure 10:**
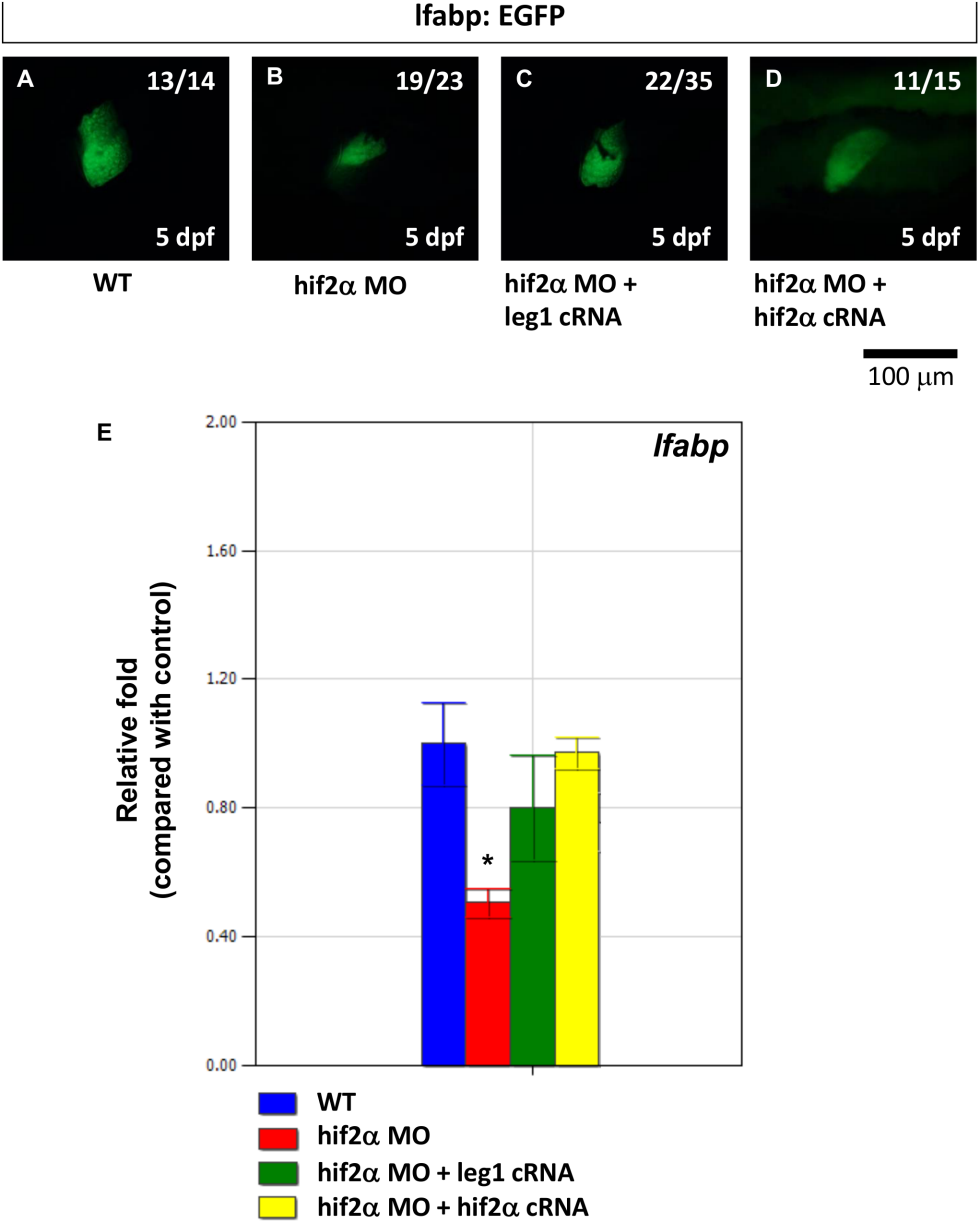
The small liver phenotype caused by *hif2-alpha* knockdown can be rescued by ectopic *leg1* expression. The expression pattern of the *lfabp* gene was examined in Tg(*lfabp*:EGFP) embryos (A) and compared with *hif2-alpha* ATG-MO-injected embryos (8 ng) (B) as well as embryos co-injected with *hif2-alpha* ATG-MO (8 ng) and *leg1* cRNA (C). (D) *lfabp* mRNA expression was detected by qPCR in wild-type embryos and compared with *hif2-alpha* ATG-MO-injected embryos, *hif2-alpha* ATG-MO and *leg1* cRNA co-injected embryos, as well as *hif2-alpha* ATG-MO and *hif2-alpha* cRNA co-injected embryos. Expression was normalized to *β-actin*.

To investigate if *hif2-alpha* regulates hepatic outgrowth through *leg1*, we conducted a rescue experiment by co-injecting *hif2-alpha* morphants with *leg1a* mRNA in Tg(*lfabp*:EGFP) transgenic fish. While *hif2-alpha* morphants revealed a small liver phenotype, we found that *hif2-alpha* morphants rescued by *leg1a* mRNA presented a significantly restored liver size at 5 dpf (Fig. 10A–10C). *lfabp* expression, as measured by qPCR, was also significantly increased in *hif2-alpha* morphants rescued by *leg1a* mRNA (Fig. 10D, *p*<0.05). Therefore, we concluded that *hif2-alpha* regulates hepatic outgrowth by regulating *leg1* expression.

### Hif2-alpha regulates *leg1* gene expression by binding to the *leg1* promoter

**Figure 11:**
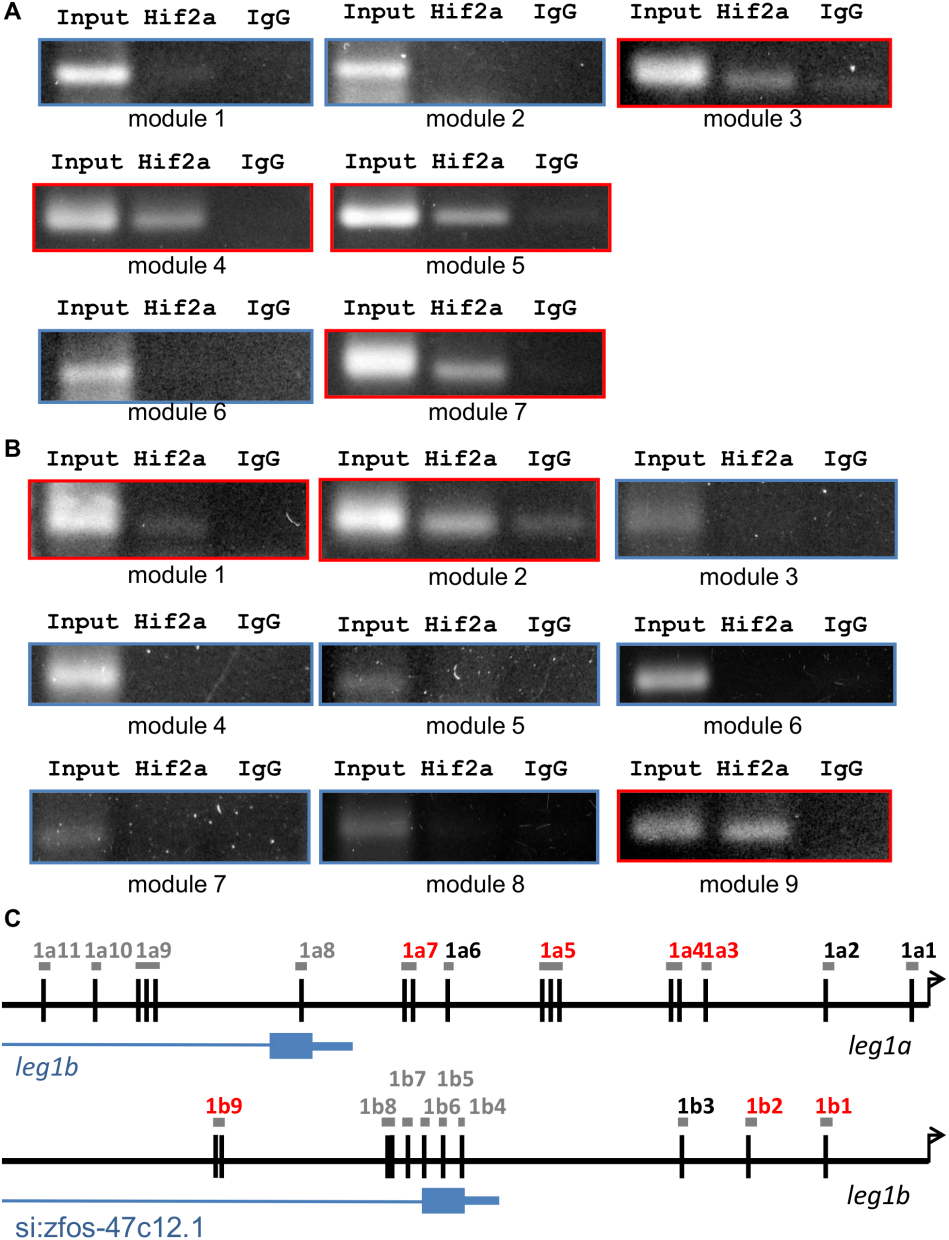
Hif2-alpha binds to *leg1a* and *leg1b* promoters. The binding of *hif2-alpha* to the promoter region of the *leg1a* (A) and *leg1b* (B) genes was examined by ChIP-PCR. Seven and nine HRE-containing modules in the promoter regions of *leg1a* and *leg1b*, respectively, are amplified from the immunocomplexes obtained by ChIP assays performed using a polyclonal antibody against anti-Hif2-alpha or a preimmune serum (IgG) as controls. (C) Schematic representation of the *leg1a* and *leg1b* promoter regions. HRE (A/G-C-G-T-G) are annotated as dark lines. The positions of the modules analyzed in the ChIP-PCR assays are shown as grey boxes. The amplified fragments in ChIP assays, including modules 3, 4, 5 and 7 in the *leg1a* promoter and modules 1, 2 and 9 in the *leg1b* promoter, are outlined in red (A, B).

Hif2-alpha is a basic helix-loop-helix transcription factor. To further identify if Hif2-alpha regulates *leg1* gene expression by directly binding to the *leg1* promoter region, we conducted ChIP-PCR to examine if Hif2-alpha binds to HREs in the *leg1* promoter. We analyzed the sequence upstream of the transcription start site for the *leg1a* and *leg1b* genes and grouped the sequences into 11 HRE modules and 9 modules, respectively. In the adult zebrafish liver, we found that Hif2-alpha binds HREs in module 3, module 4, module 5, and module 7 in the *leg1a* promoter. Hif2-alpha can also bind HREs in module 1, module 2, and module 9 in the *leg1b* promoter (Fig. 11). These results suggest that Hif2-alpha regulates *leg1* gene expression by binding to the HRE modules in the *leg1* promoter.

### The involvement of the FGF, HGF, and Wnt pathways in hepatic outgrowth regulated by Hif2-alpha

**Figure 12:**
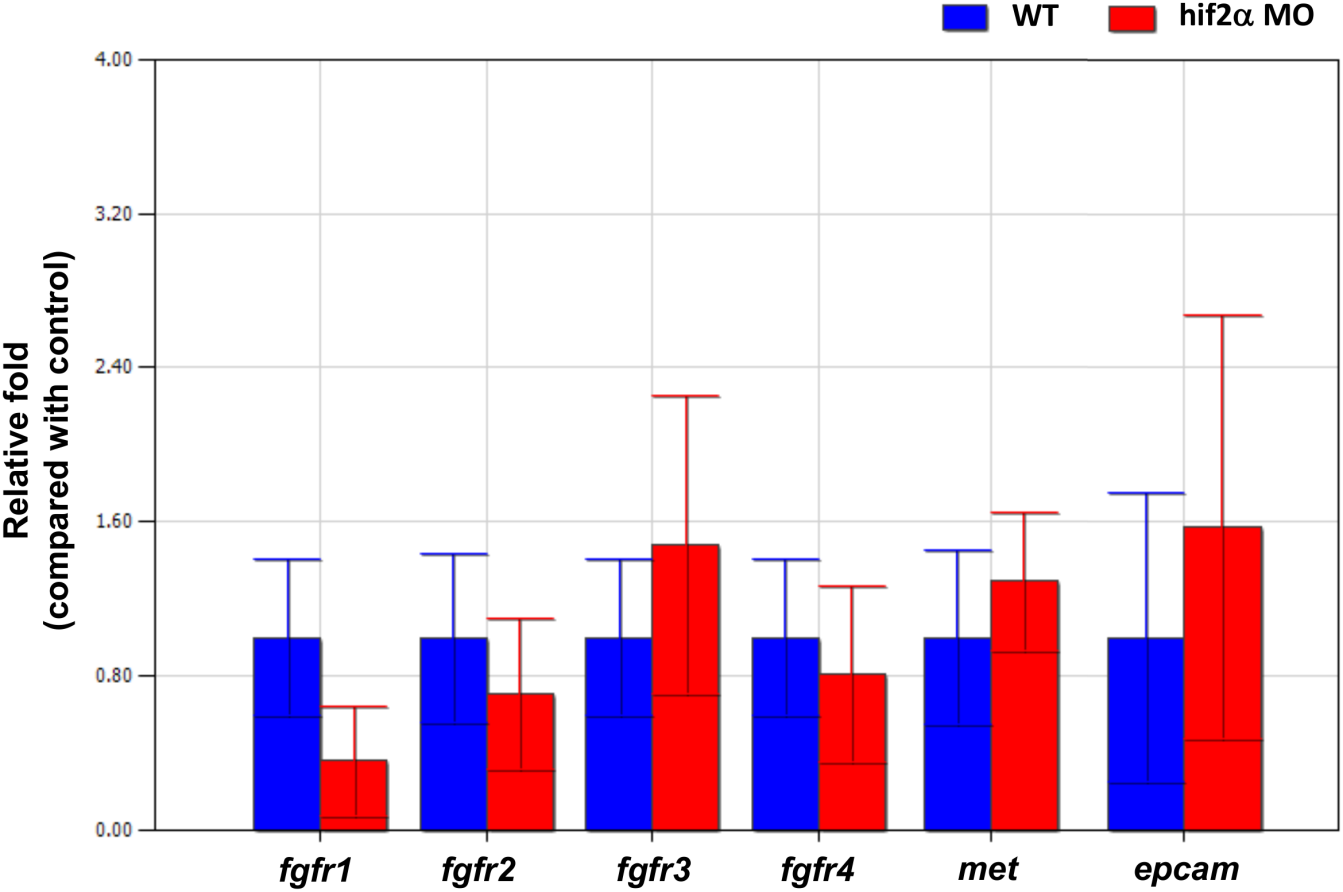
The regulation of hepatic outgrowth by *hif2-alpha* in zebrafish embryos is not through the FGF, HGF, or Wnt pathways. Gene expression of *fgfr1*, *fgfr2*, *fgfr3*, *fgfr4*, *met*, and *epcam* was examined in wild-type embryos and compared with *hif2-alpha* ATG-MO-injected embryos at 3–5 dpf by qPCR. Expression was normalized to *β-actin*.

Several factors in the FGF, HGF, and Wnt pathways have been demonstrated to be involved in liver development in zebrafish [45], [51], [52]. To investigate if Hif2-alpha regulates hepatic outgrowth through the FGF, HGF, and Wnt pathways, we checked the gene expression of *fgfr1*, *fgfr2*, *fgfr3*, *fgfr4* (the FGF receptors for hepatic development in zebrafish), *c-met* (the HGF receptor for hepatic development in zebrafish), and *epcam* (the Wnt derepressor for hepatic development in zebrafish). We found no significant change of those genes in *hif2-alpha* morphants compared to wild-type embryos at 5 dpf by qPCR (Fig. 12). This result indicated that the regulation of hepatic outgrowth by *hif2-alpha* was not directly related to the FGF, HGF, and Wnt pathways.

### CoCl_2_ treatment increased the expression of the target genes of *Hif1-alpha* rather than those of *Hif2-alpha*

**Figure 13:**
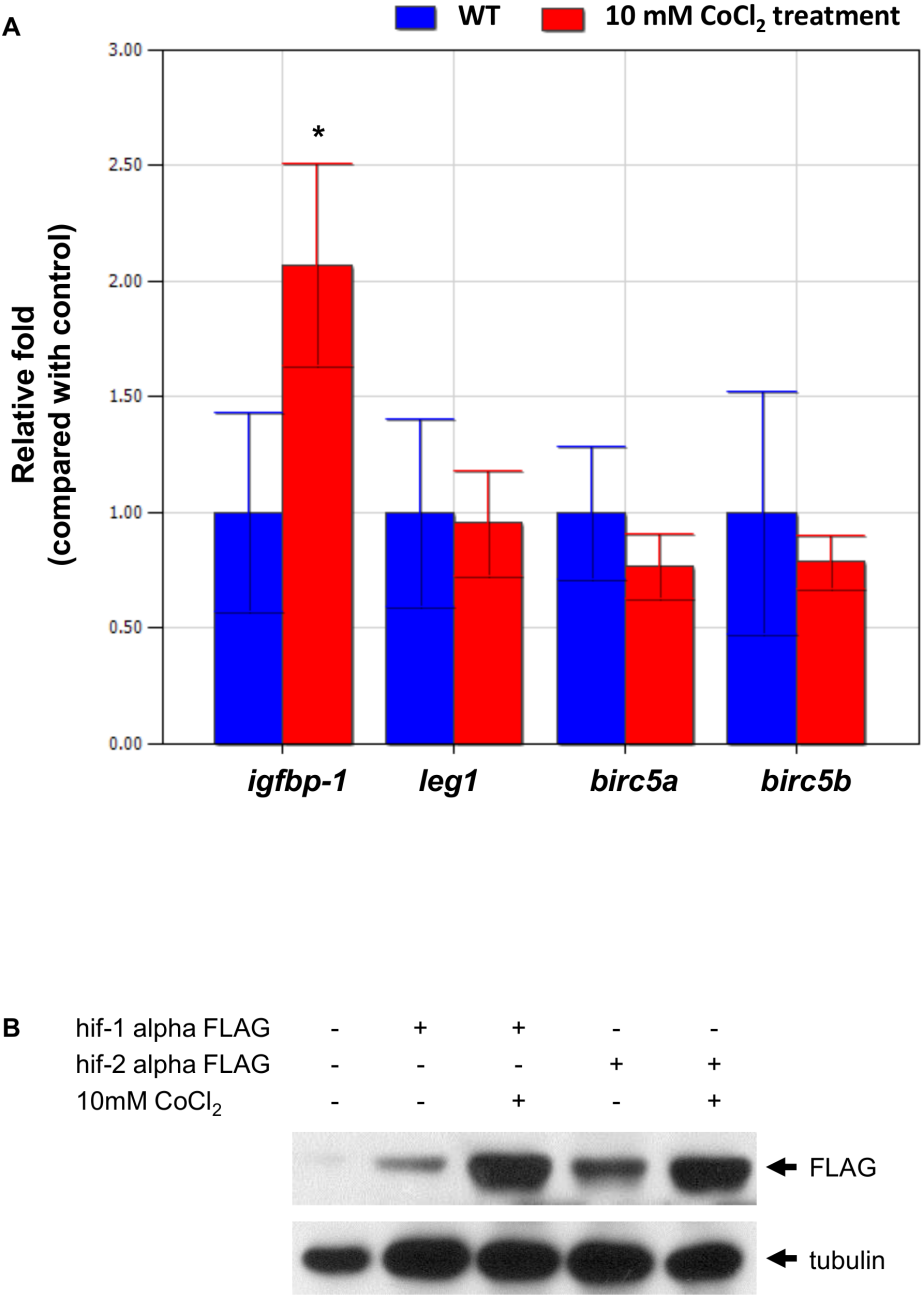
CoCl_2_ treatment increased the expression of the target genes of Hif1-alpha rather than those of Hif2-alpha. (A) Gene expression of *igfbp-1*, *leg1*, *birc5a*, and *birc5b* mRNA without (control, in blue) or with 10 mM CoCl_2_ treatment (red) was examined in zebrafish embryos at 72 hpf by qPCR. Expression was normalized to *β-actin*. *indicates a significant difference (*p*<0.05) between control embryos and CoCl_2_-treated embryos by an unpaired *t-test*. The experiment was performed in triplicate, and error bars indicate the standard deviation. (B) The Flag-protein expression levels were performed by western blot and were normalized to tubulin.

Because we demonstrated that Hif2-alpha regulates gene expression by directly binding to the sequences upstream of both *leg1a* and *leg1b*, a hypoxia-mimic treatment should enhance the stability of Hif2-alpha and up-regulate *leg1* gene expression. Here, CoCl_2_ was used to mimic hypoxic conditions [53]. We incubated zebrafish embryos in 10 mM CoCl_2_ from 48 hpf to 72 hpf. While the up-regulation of *igfbp-1* expression [48] was evident, we did not detect a significant difference in the expression of the *leg1*, *birc5a* and *bir5b* genes. (Fig. 13A) (*birc5a* and *birc5b* are two proven direct targets of Hif2-alpha [33]). These results suggest that a selective up-regulation of the downstream genes of *hif1-alpha* but not those of *hif2-alpha* in zebrafish embryos exists with hypoxia-mimic treatment.

To further prove the selective up-regulation of *Hif*-target genes by CoCl_2_, we investigated the stability of Hif1-alpha and Hif2-alpha proteins in zebrafish embryos. We injected *Hif1-alpha-FLAG* and *Hif2alpha-FLAG* mRNA into fertilized eggs, respectively, incubated zebrafish embryos in 10 mM CoCl_2_ from 48 hpf to 72 hpf, and extracted the protein from the embryos. We found that the stability of Hif1-alpha protein was increased after CoCl_2_ treatment (5.03-fold); in contrast, the stability of Hif2-alpha protein only increased slightly after CoCl_2_ treatment (1.98-fold) (Fig. 13B).

### HRE clusters are found upstream of the *leg1* gene in teleosts

**Figure 14:**
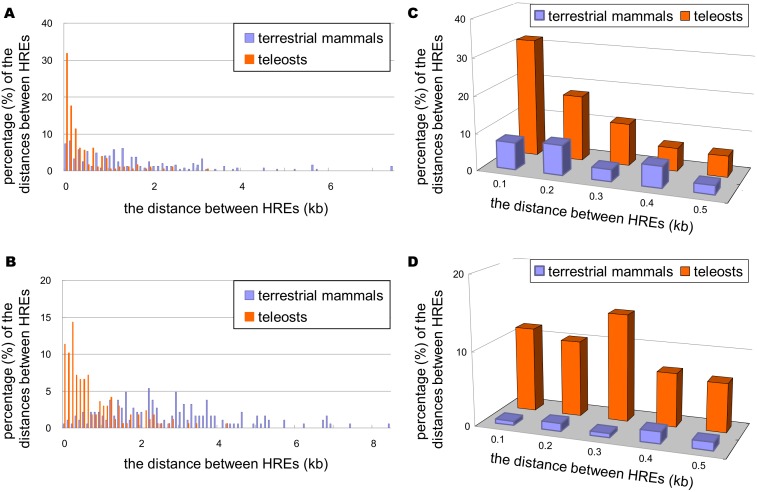
Distribution of HRE clustering. The distances between HRE for the first neighbored HRE (A, B) and the second neighbored HRE (C, D) were measured, and the distributions of the distance are shown in 100 bps bins.

A homotypic binding cluster of transcription factors had been shown to be important in the tissue-specific expression of regulated genes and crucial for embryonic development. Because we showed that some HREs are bound by Hif2-alpha using ChIP-PCR, we wanted to examine if HRE clusters exist in the regulatory sequences of *leg1*. We mapped HREs in the upstream, potential regulatory sequences (relative to the translational start site, 10 kbps) of *leg1* genes in terrestrial mammals (34 species) and teleosts (7 species). We analyzed the distribution of the distances between HREs and their first neighbors and second neighbors. We found that in the upstream sequences of the teleost *leg1 g*ene, HREs tend to cluster and form potential high-density Hif-binding regions. However, this was not observed in the upstream sequences of the terrestrial mammalian *leg1* gene (Fig. 14A–D). If we used 500 bps as the window size and counted the number of binding sites inside this window, we found that 86% of teleost sequences (including module 3, module 4, module 5, and module 7 of the *leg1a* gene of zebrafish) contain more than or equal to 3 HREs in comparison with 18% of terrestrial mammalian sequences (*p*<0.01) (Table 1, Table S3). Therefore, we concluded that high-density HREs in upstream, potential regulatory sequences are observed in teleosts but not in terrestrial mammals.

**Table 1:** HRE cluster detection in the upstream sequences of animals.

Category^a^	Species^b^	Total species^c^	Ratio^d^
Mammalia	6	34	18%
Aves	2	5	40%
Reptilia	1	3	33%
Teleost	6	7	86%

a the category of the animals.

b the number of species of which upstream sequences contain no less than three HREs in a window of 500 bps.

c the species number in a category.

d the ratio of **^b^** ^/**c**^.

## Discussion

There are three major findings in this study. First, we present the first evidence that *hif2-alpha* plays an important role in embryonic hepatic outgrowth. Second, we show that the ability of *hif2-alpha* to drive hepatic outgrowth is mediated by *leg1*. Third, the *hif2-alpha-leg1* axis may be an adaptation in teleosts that is not observed in terrestrial mammals. We provided the evidence that *hif2-alpha* is required for hepatic outgrowth but not hepatoblast specification in the zebrafish embryo. Moreover, the lack of Hif2-alpha significantly decreased *leg1* expression. Exogenous *leg1* mRNA can restore the liver outgrowth defect caused by *hif2-alpha* knockdown. Furthermore, Hif2-alpha protein molecules can bind to HRE in the promoter sequence upstream of the *leg1* gene. These observations suggest that leg1 is the gene that is downstream of *hif2-alpha* and that *hif2-alpha* regulates hepatic development by acting on *leg1*. Furthermore, high-density HREs can be found in the potential regulatory sequences of *leg1* in teleosts but not in terrestrial mammals.

### The *Hif2-alpha-leg1* axis during embryonic development in the zebrafish

The phenotype of the gene knockdown of *leg1* and *hif2-alpha* supports the idea that *leg1* is the gene that is downstream of *hif2-alpha*. Knockdown of *leg1* has been shown to block liver expansion; however, liver initiation is not affected [26]. These results are similar to our data detailing the knockdown of *hif2-alpha.* We also found that knockdown of *hif2-alpha* revealed a smaller exocrine pancreas and intestine (Fig. 6C, D). Again, this is similar to what was observed with the knockdown of *leg1* [26]. The likelihood of the *leg1* gene as a downstream target of *hif2-alpha* is also supported by ChIP-PCR, where we demonstrated that Hif2-alpha directly binds to HRE in the promoter region of both *leg1a* and *leg1b*.

Numerous genes have been revealed to be important during the outgrowth stage, including *alr* [54], *uhrf1* [43], *hdac3* [55], *gdf11* [55], *def* [56], [57], *delta113p53* [57], *pes* [58], *mypt1* [59], *npo* [60], *c-met* [51], [61], *grnA* [51], *bms1l* [62], and *leg1* [26]. In our study, the *Hif2-alpha-leg1* axis regulates hepatic outgrowth but does not play a role in liver specification and differentiation. In addition to *leg1* [26], both *def* [56], [57] and *npo* [60] affect hepatic outgrowth but not liver specification and differentiation. It is possible that multiple factors are required for cell proliferation and the formation of liver morphology during the hepatic outgrowth and expansion phase. These factors may interact with each other through functional association or physical interaction, and there is little redundancy in this interaction network. A lack of any factor would abolish hepatic outgrowth to varying extents. In this context, *grnA* had been shown to regulate hepatic outgrowth by modulating *c-met* gene expression [51]. Therefore, it is of interest to identify whether the *grna*-*c-met* axis [51] can exert its influence on liver development through a pathway other than the *hif2*-*leg1* axis. Further studies are required to analyze the role of each factor and their interdependence during hepatic outgrowth. It is important to note that the rescue of *hif2-alpha* morphants by *leg1* cRNA can restore the liver to a partial wild-type volume. This is consistent with the concept mentioned above, suggesting that *leg1* is one of the multiple factors involved in hepatic outgrowth. Additionally, it is possible that *hif2-alpha* may control a distinct mechanism of hepatic outgrowth when *leg1* is one of the downstream “executors” of *hif2-alpha*.

In our previous study, we demonstrated that the knockdown of *hif2-alpha* caused apoptosis in neuronal cells in the zebrafish embryo. In neuron cells, Hif2-alpha induces the gene expression of survivin (*birc5A* and *birc5B*), which regulates cell apoptosis. Interestingly, a lack of Hif2-alpha or survivin causes the cell to maintain a progenitor cell state and to continue to proliferate while inducing apoptosis in neuron cells [33]. In this study, the knockdown of *hif2-alpha* reduces cell proliferation but does not result in apoptosis in the developing liver of zebrafish embryos. Moreover, *Hif2-alpha* affects neuronal development as early as 1 dpf [33] and influences hepatic outgrowth starting at 3 dpf. This raises the possibility that *Hif2-alpha* employs various temporal regulatory mechanisms when comparing development in neurons and in the liver.

Hif2-alpha is a transcription factor, and its functions are restricted to the cell where it is expressed. However, in adult zebrafish, the Hif2-alpha-inducible *leg1* gene encodes a secretory protein that exists in the liver, head, tail, gut and serum; however, most of the *leg1* transcripts are found in adult livers [26]. Interestingly, Hif-alpha can also induce the gene expression of other secretory proteins, such as insulin growth factor–binding protein-1 (IGFBP-1) [63], [64], insulin growth factor–binding protein-2 (IGFBP-2) [65], and Galectin-1 [66]. The knockdown of *IGFBP-1* in zebrafish has been shown to cause growth retardation and developmental delays [63]. IGFBP-2 enhances tumor angiogenesis by promoting angiogenesis and facilitating the induction of VEGF-A in endothelial cells [65]. Galectin-1 suppresses T cell-mediated cytotoxic immune responses and promotes tumor angiogenesis [66]. Therefore, we speculate that there are additional Hif-inducible genes that encode secretory proteins that may play crucial roles during embryonic development and carcinogenesis.

### The evolutionary implications of homotypic clusters of Hif-binding sites

The clustering of transcription factor binding sites is observed in higher eukaryotes [30], [67]. Enhancers containing the homotypic clusters of transcription factor binding sites were shown to enable a tissue-specific expression pattern in *Drosophila*, zebrafish (POU3F2) and mouse embryos (ETS, E2F4, NRF1, POU3F2), thereby suggesting an important role for the homotypic clusters of transcription factor binding sites during embryonic development [68]–[70].

In our previous study, we identified the unusually clustered aryl hydrocarbon receptor response element (AHRE) in the upstream regulatory sequences of the *CYP3* genes in teleosts but not in terrestrial mammals [71]. Here, we also discovered high-density HREs in the potential upstream regulatory sequences of *leg1* genes in teleosts but not in terrestrial mammals. We speculate that Hif2-regulated liver development through *leg1* is an adaptation observed in teleosts as a result of their aquatic environment. It should be noted that the importance of the homotypic high-density transcription factor binding sites is based on an assumption that high-density homotypic DNA elements are more likely to be bound by corresponding transcription factors, thus recruiting chromatin-modification proteins and enabling a transcription-capable status for the regulated genes. Whether this assumption holds will depend on the investigation of the ability of Hif2-alpha to regulate *leg1* gene expression in terrestrial mammals and teleosts.

### Differential stability and different targets of Hif-alpha molecules reflect the diverse regulation of biological functions

We found that Hif2-alpha but not Hif1-alpha is required for liver development. Although Hif1-alpha and Hif2-alpha can target the same genes, they also each have their own respective unique target genes, thereby reflecting the different roles these genes play in the cellular homeostasis in hypoxia/anoxia and diseases [72]. In zebrafish embryos, Hif2-alpha is required for neuron development [33]. However, Hif1-alpha controls neural crest chemotaxis and the epithelial to mesenchymal transition by inducing the expression of the chemokine receptor Cxcr4 and Twist [30].

In addition to this difference in their target genes, the presence of Hif2-alpha protein in cells was reported not to depend on the hypoxia state; nevertheless, Hif1-alpha is ready to enhance the hypoxia-induced stability [73]–[75]. In our study, in the zebrafish embryo, the Hif1-alpha target gene can be induced upon treatment with CoCl_2_. We also demonstrated that the stability of Hif1-alpha is more significantly increased in zebrafish embryos than the stability of Hif2-alpha after treatment with CoCl_2_. CoCl_2_ has long been used to mimic hypoxia and induces Hif1-alpha expression in rabbit hepatocytes [76]. CoCl_2_ also binds to the oxygen-dependent degradation (ODD) domain of Hif2-alpha, thereby preventing its association with the von Hippel-Lindau protein [77]. In bovine arterial endothelial cells, treatment of CoCl_2_ did not change the stable presence of Hif2-alpha in the cell [78]. Interestingly, a similar observation was also detected in primary mouse hepatocytes in a 1% oxygen environment [79]. Our observations are consistent with the results of the previous studies mentioned above, indicating the differential stability and control upon Hif-alpha molecules during the regulation of the various aspects of embryonic development.

### The regulation of hepatic outgrowth by *hif2-alpha* in zebrafish embryos is not through the FGF, HGF, or Wnt pathways

In mammals, FGF signaling is important for hepatic specification [7]. The fibroblast growth factors (FGF) expressed by the cardiac mesoderm induce the formation of the liver from the foregut endoderm. In zebrafish, FGF signaling is not only essential for hepatoblast specification [13] but also necessary for hepatic outgrowth [45]. When FGF signaling is blocked at 22 hpf, the gene expression levels of hepatoblast markers, such as *hhex* and *prox1*, are depressed [13]. The liver size is reduced in Tg(*lfabp:dnfgfr1-egfp*) zebrafish, which expresses a domain-negative fgfr1-egfp at 5 dpf [45]. We surveyed the gene expression levels of *fgfr1*, *fgfr2*, *fgfr3*, *fgfr4* in *hif2-alpha* morphants compared to wild-type embryos and demonstrated that the gene expression of the FGF receptors was not affected when *hif2-alpha* was knocked down.

HGF signaling is essential for hepatoblast proliferation and hepatocyte differentiation in mammals [80]. In HGF^−/−^ mutant mice [81] and cMet^−/−^ (the HGF receptor) mutant mice, the liver was undeveloped and smaller [82]. In zebrafish, knockdown of *met* affected hepatic outgrowth but not hepatoblast specification [61]. Our study revealed that *met* expression was not changed significantly when the *hif2-alpha* was knocked down in zebrafish at 5 dpf.

In mammals, Wnt signaling plays two opposing roles in liver development. In endoderm patterning (the stage before hepatoblast specification), Wnt signaling represses the liver cell fate. After the hepatoblast specification stage, Wnt signaling induces hepatocyte differentiation and outgrowth [1]. In zebrafish, Wnt signaling is important for hepatic specification, hepatocyte differentiation and outgrowth [3], [52]. The liver was smaller, and the expression of the hepatoblast markers, *hhex* and *prox1*, were decreased in *wnt2bb* mutant zebrafish [83]. Overexpression of *epcam* (a Wnt derepressor) resulted in a larger liver in zebrafish embryos [52]. In this study, we proved that the expression level of *epcam* was not altered significantly in *hif2-alpha* morphants compared to wild-type embryos.

These observations suggest that the FGF, HGF and Wnt pathways are upstream of the Hif pathway or that the FGF, HGF and Wnt pathways and the Hif pathway are parallel to each other in the regulation of hepatic growth.

### EPO is not regulated by *hif2-alpha* in zebrafish embryos

Erythropoietin (EPO) is necessary for erythropoiesis and is regulated by *hif2-alpha* in mice. A previous study showed that EPO production was suppressed in *hif2-alpha* mutant mice [49]. The epo expression level was also down-regulated in *hif1-alpha* mutant mice [84]. In zebrafish, *epo* expression was up-regulated in *vhl* mutants at 7.5 dpf [85], implying that *epo* was up-regulated by the more stable Hif protein. In this study, we examined the expression levels of *epo* in *hif2a-alpha* morphants, and the results revealed that *epo* gene expression was not changed when *hif2-alpha* was knocked down (Fig. 7). Because the gene expression pattern data revealed that both *hif1-alpha* and *hif2-alpha* were expressed in the liver (Fig. 1), it is possible that other family members of Hif (eg, *hif1-alpha*) can regulate *epo* gene expression in the zebrafish embryo.

### Expression of genes responsible for lipid metabolism can be regulated by *hif2-alpha*

Lipid metabolism is comprised of several processes, including lipogenesis, lipid oxidation, and cholesterol synthesis. In lipogenesis, *fasn* gene expression has been shown to be suppressed in hypoxic HepG2 cells in a *hif2-alpha*-dependent manner [50]. In lipid oxidation, the gene expression levels of *cpt1* (oxidation) have been shown to be down-regulated in mutant mice containing a stable form of Hif2-alpha. In cholesterol synthesis, hypoxia can stimulate the degradation of HMG-CoA reductase and increase the gene expression of HMG-CoA synthase and HMG-CoA reductase in CHO-7 cells [86]. Recently, the loss of Von Hippel-Lindau (VHL) was shown to increase the cholesterol level via Hif2-alpha [87].

In this study, we reported that the expression levels of the genes involved in lipogenesis (*acc1*, and *fasn*) were not changed in *hif2-alpha* morphants (Fig. 7). We also demonstrated that the expression levels of the genes involved in lipid oxidation (*cpt1* and *echs1*) were down-regulated in *hif2-alpha* morphants compared to wild-type embryos. Moreover, the expression levels of the genes responsible for cholesterol synthesis (HMG-CoA synthase (*hmgcs1*) and HMG-CoA reductase (*hmgcrb*)) were shown to be up-regulated in *hif2-alpha* morphants compared to wild-type embryos. While *hif2-alpha* might be involved in the regulation of lipid oxidation and cholesterol synthesis in the zebrafish embryo, it is interesting to note that the trend of this influence is different between mammals and zebrafish embryos. Recently, more experiments have shown that lipid/cholesterol regulation is important for embryonic development [88], [89]. The inclusion of metabolites and a more systematic approach for identifying the involvement of the Hif pathway shall certainly help to resolve the current inconsistency between different model organisms.

## Conclusion

We demonstrated that *Hif2-alpha* plays an essential role in the embryonic development of hepatic outgrowth in zebrafish through directly controlling *leg1* gene expression. We also speculated that the Hif2-alpha-leg1 axis may be an adaptation in teleosts because high-density Hif-binding sites in the potential upstream regulatory sequences are observed in teleosts but not in terrestrial mammals.

## Supporting Information

Figure S1 **Whole-mount in** **situ hybridization was performed with sense probes of** ***hif1-alpha*** **,** ***hif2-alpha*** **and** ***hif3-alpha*** **.** The expression patterns of *hif1-alpha* (A–D), *hif2-alpha* (E–H) and *hif3-alpha* (I–L) were assessed with sense probes by WISH in zebrafish embryos at 2–5 dpf. WISH, whole-mount in situ hybridization. dpf, days post-fertilization.
(TIF)

Figure S2 **Knockdown of** ***hif2-alpha*** **reduced cell proliferation in the trunk and tail of zebrafish embryos.** Cell apoptosis in wild-type embryos (A) and *hif2-alpha* ATG-MO-injected embryos (B) at 4 dpf by pH3 staining.
(TIF)

Figure S3 **Knockdown of** ***hif2-alpha*** **did not induce cell apoptosis in hepatocytes in the zebrafish embryos.** Cell apoptosis in Tg(*lfabp*:EGFP) embryos (A, B, C) and *hif2-alpha* ATG-MO-injected Tg(*lfabp*:EGFP) embryos (D, E, F) at 4 dpf by TUNEL assay. A positive control using Tg(*lfabp*:EGFP) embryos with DNaseI treatment is also shown (G).
(TIF)

Figure S4 ***Leg1*** **is not required for liver specification in zebrafish embryos.** Liver specification in *leg1* morphants was detected through the expression of the *hhex* and *prox1* genes. The expression of embryonic liver specification genes, *hhex* (A, B, E, F) and *prox1* (C, D, G, H), were examined at 30 hpf (A–D) and 55 hpf (E–H) in wild-type and *leg1* ATG-MO-injected embryos by WISH.
(TIF)

Table S1 **Primer sequences employed in the qPCR experiments.**
(DOC)

Table S2 **Primer sequences employed in the ChIP-PCR experiments.**
(DOC)

Table S3 **The summary of HRE clusters in the upstream sequences of** ***leg1*** **comparing with ChIP-PCR assay.**
(DOC)
