# Supplementary figures and images for: Hypoxia-Inducible Factor 2 Alpha Is Essential for Hepatic Outgrowth and Functions via the Regulation of leg1 Transcription in the Zebrafish Embryo
Source: PLoS One. 2014 Jul 7;9(7):e101980. doi: 10.1371/journal.pone.0101980 (PMC4084947; doi:10.1371/journal.pone.0101980)

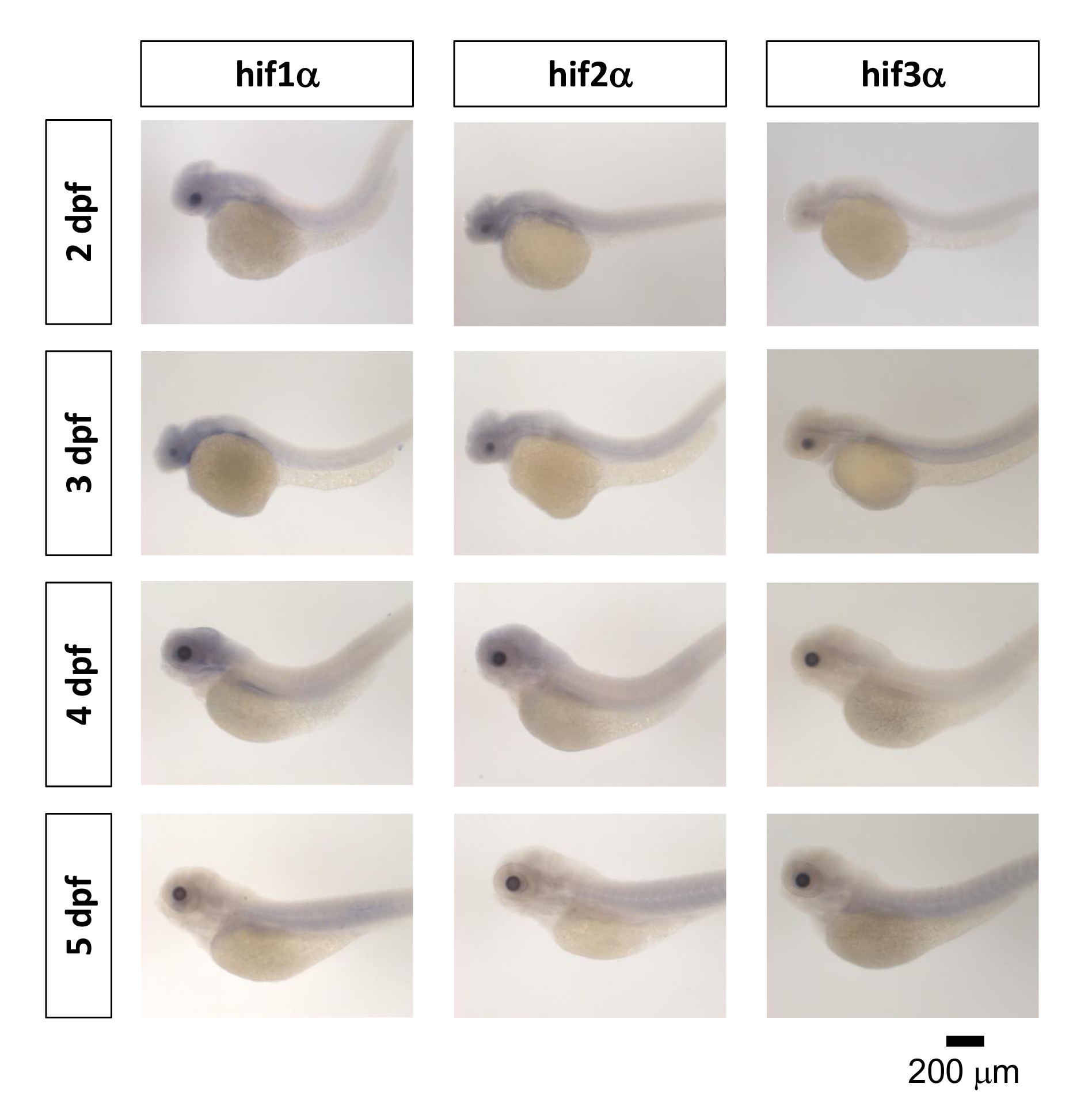

Supplement: Figure S1 — Whole-mount in situ hybridization was performed with sense probes of hif1-alpha , hif2-alpha and hif3-alpha . The expression patterns of hif1-alpha (A–D), hif2-alpha (E–H) and hif3-alpha (I–L) were assessed with sense probes by WISH in zebrafish embryos at 2–5 dpf. WISH, whole-mount in situ hybridization. dpf, days post-fertilization. (TIF) [file pone.0101980.s001.tif]

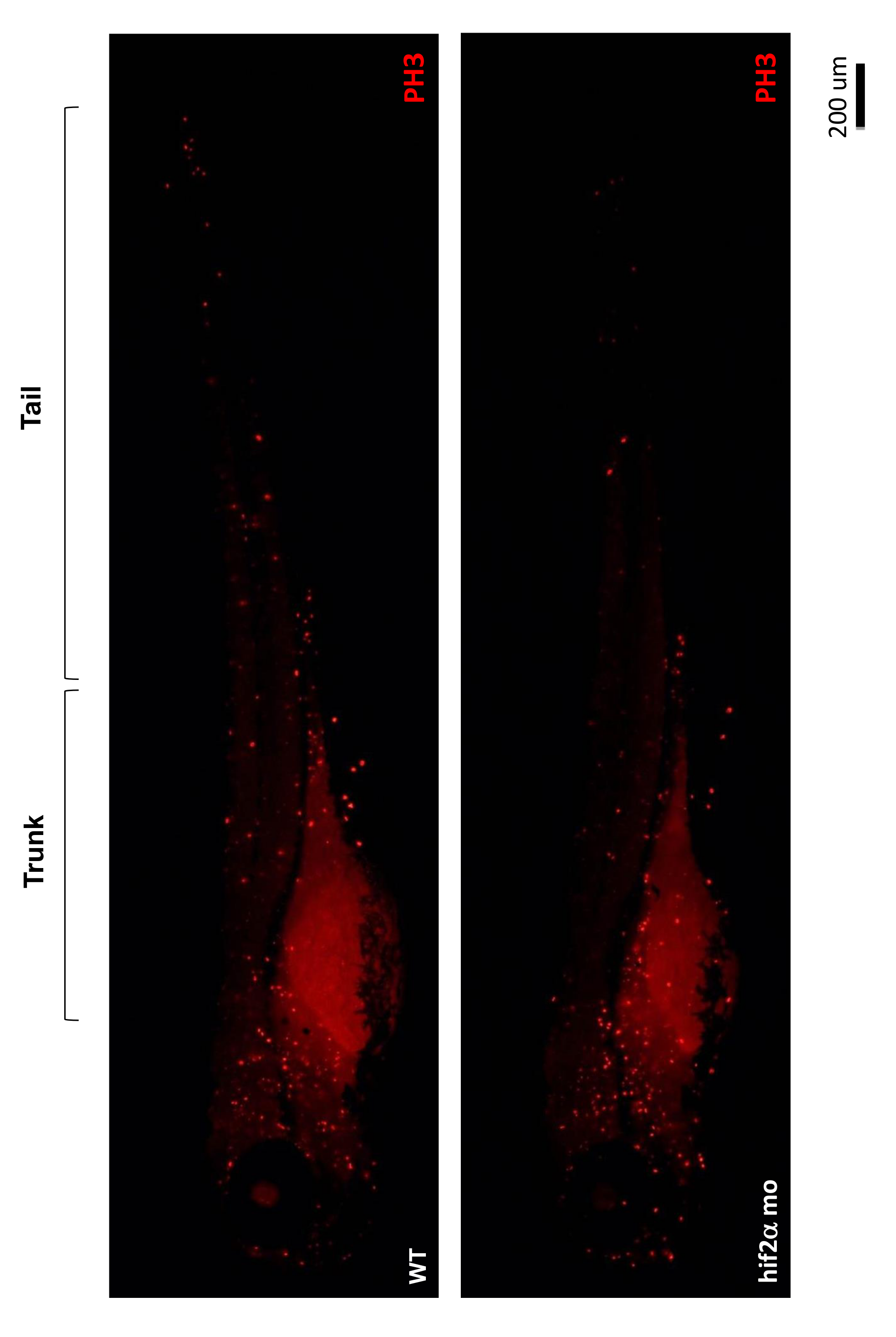

Supplement: Figure S2 — Knockdown of hif2-alpha reduced cell proliferation in the trunk and tail of zebrafish embryos. Cell apoptosis in wild-type embryos (A) and hif2-alpha ATG-MO-injected embryos (B) at 4 dpf by pH3 staining. (TIF) [file pone.0101980.s002.tif]

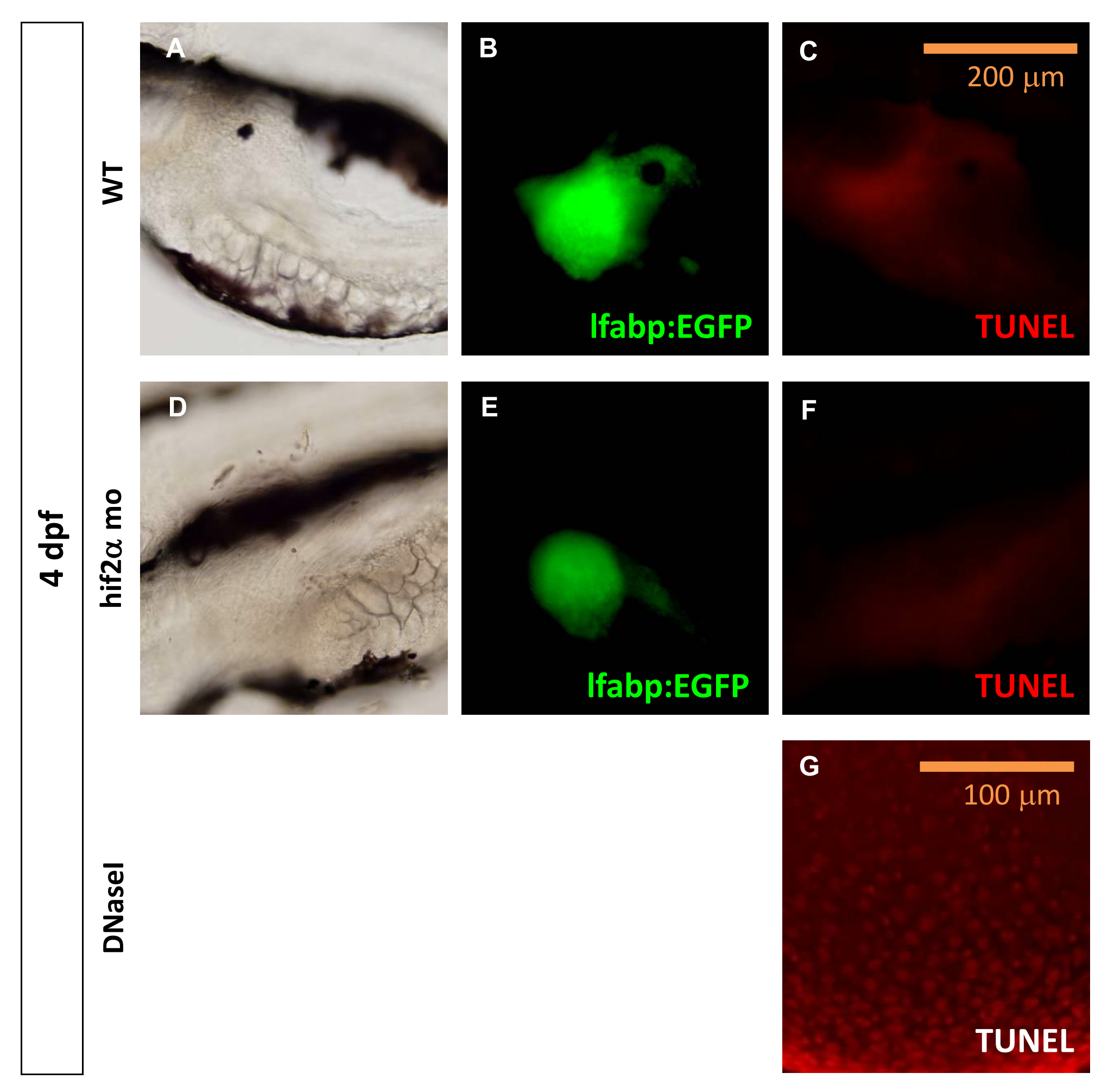

Supplement: Figure S3 — Knockdown of hif2-alpha did not induce cell apoptosis in hepatocytes in the zebrafish embryos. Cell apoptosis in Tg(lfabp:EGFP) embryos (A, B, C) and hif2-alpha ATG-MO-injected Tg(lfabp:EGFP) embryos (D, E, F) at 4 dpf by TUNEL assay. A positive control using Tg(lfabp:EGFP) embryos with DNaseI treatment is also shown (G). (TIF) [file pone.0101980.s003.tif]

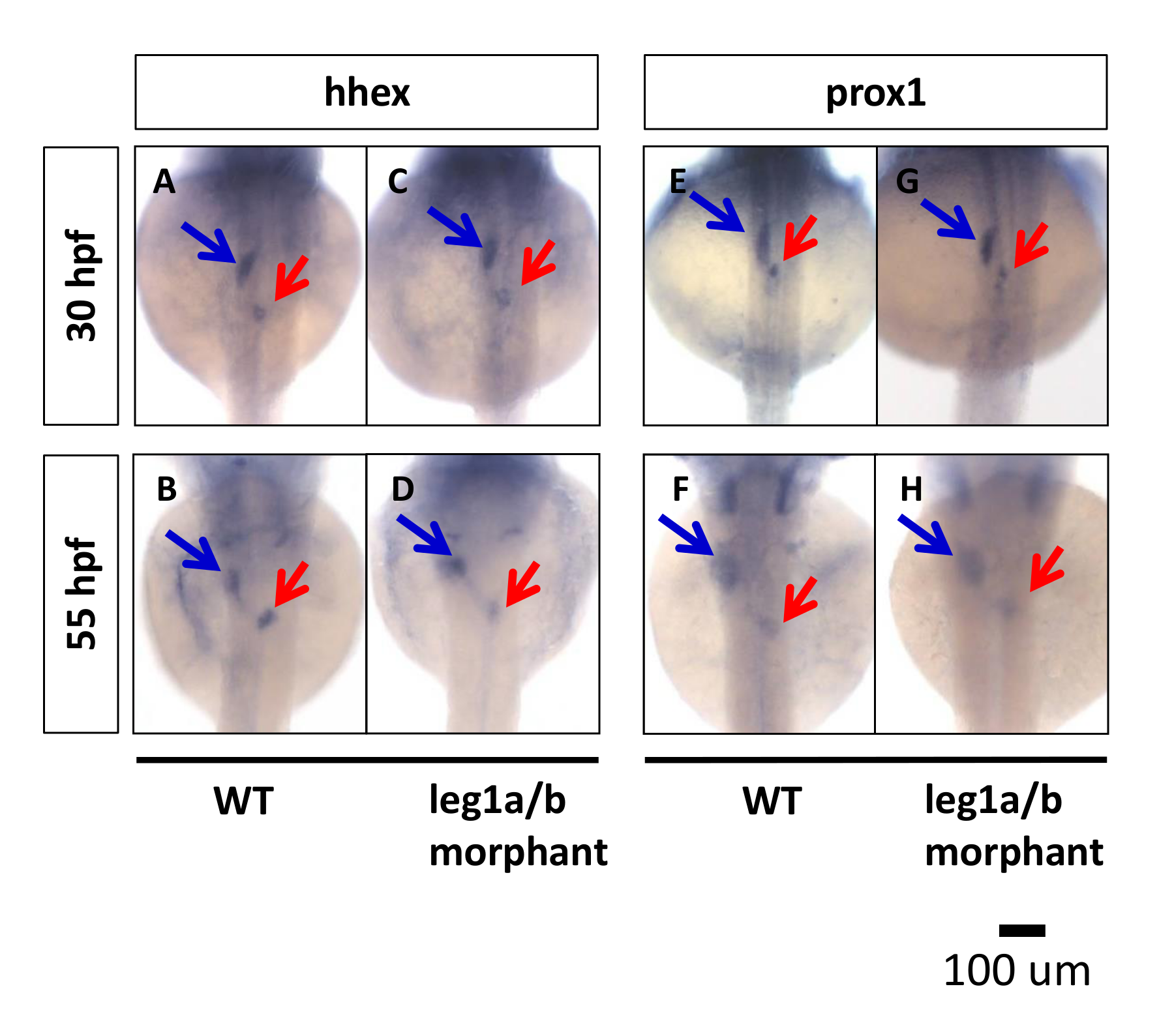

Supplement: Figure S4 — Leg1 is not required for liver specification in zebrafish embryos. Liver specification in leg1 morphants was detected through the expression of the hhex and prox1 genes. The expression of embryonic liver specification genes, hhex (A, B, E, F) and prox1 (C, D, G, H), were examined at 30 hpf (A–D) and 55 hpf (E–H) in wild-type and leg1 ATG-MO-injected embryos by WISH. (TIF) [file pone.0101980.s004.tif]
