# Supplementary material for: Hypoxia-Inducible Factor 2 Alpha Is Essential for Hepatic Outgrowth and Functions via the Regulation of leg1 Transcription in the Zebrafish Embryo
Source: PLoS One. 2014 Jul 7;9(7):e101980. doi: 10.1371/journal.pone.0101980 (PMC4084947; doi:10.1371/journal.pone.0101980)
Supplement: Table S1 — Primer sequences employed in the qPCR experiments. (DOC) [file pone.0101980.s005.doc]

**Table S1. Primer sequences employed in the qPCR experiments.**

| **Name^a^** | **Forward^b^** | **Reverse^c^** |
| --- | --- | --- |
| *lfabp* | TAAGCTGACAGCGTTTGTGAAGG | AGATGCGTCTGCTGATCCTCTTGT |
| *fabp10a* | CACCTCCAAAACTCCTGGAA | TTCTGCAGACCAGCTTTCCT |
| *leg1* | TCTGGTGGATTTGTGGCG | GAGCATGATGATCGGTGT |
| *igfbp-1* | GTCAATGAAGGCAGCTCCAC | TCTTGCGTATCGCGTTGACT |
| *birc5a* | GTGGCCACGCGATTGAA | CAGTCTGGATGATCTGGTTTCTCTT |
| *birc5b* | GGAGCGACTTCGCATCTACAT | ACCTCATCACGAAAGTAGGCAATC |
| *fgfr1* | CTTGCTTTGCTCAGGGACTC | CCGCATGTAGCTTCTTCTCC |
| *fgfr2* | GCACAAGCTCACCAAACAGA | TCAGGGAGGTCGTATTCTGG |
| *fgfr3* | ATCGTATGGCAAAAAGACGG | CAGCGAATCGATGACTTTGA |
| *fgfr4* | AGGGTGCTGGTGTCAATTTC | TTAGGTCCATCCGAGAATGC |
| *met* | AGGAACAGGCCCAGAAAGAT | TTGTGGCTGAAACACTCTGC |
| *epcam* | CGGAGACCTATTGGGTTCGTG | GCCTGCCATCCTTGTCATACTG |
| *acc1* | GCATAGGGCAGGTTTTACCA | GCCATCATACGAGAGCAACA |
| *fasn* | GAGAAAGCTTGCCAAACAGG | GAGGGTCTTGCAGGAGACAG |
| *hmgcs1* | CTCACTCGTGTGGACGAGAA | GATACGGGGCATCTTCTTGA |
| *hmgcra* | CTGAGGCTCTGGTGGACGTG | CGCCGCAGCTACGATGTTGGCG |
| *hmgcrb* | GCCTGTTAGCCGTCAGTGGA | CGGCCTTTGACCACTCGTGCCG |
| *cpt1* | CTGCGATACCTCCTCAAAGC | TACAGGCAGATGTGGCAGAG |
| *echs1* | AGATGCAGAATCGAACCTTCCAA | CGGAGATAGCAAACTCACATCCTCCG |
| *epo* | CTCTTTGCCTTACTGCTGATG | CATAGCAGCCTCTGCATC |
| *beta-actin* | CCCCGAGAGGACAACAATGTA | TGAGGAGGGCAAAGTGGTAAA |

**^a^**the gene symbol

**^b^**the forward primer sequence orientation is 5’ end to 3’ end

**^c^**the reversed primer sequence orientation is 5’ end to 3’ end
