# Supplementary material for: Hypoxia-Inducible Factor 2 Alpha Is Essential for Hepatic Outgrowth and Functions via the Regulation of leg1 Transcription in the Zebrafish Embryo
Source: PLoS One. 2014 Jul 7;9(7):e101980. doi: 10.1371/journal.pone.0101980 (PMC4084947; doi:10.1371/journal.pone.0101980)
Supplement: Table S2 — Primer sequences employed in the ChIP-PCR experiments. (DOC) [file pone.0101980.s006.doc]

**Table S2. Primer sequences employed in the ChIP-PCR experiments. (DOC)**

| **Module^a^** | **Forward^b^** | **Reverse^c^** |
| --- | --- | --- |
| leg1a-1 | AGGCTCATAACTGAATCGA | ACACACCCACTGCACAAA |
| leg1a-2 | GTTGGGATTAGCTAAATACACC | AAACACGAATGCCCCATT |
| leg1a-3 | GAAAGTCTGAATGTGCGA | TATACAAGGCCTGGGTAA |
| leg1a-4 | CTCATGTTATATCAGGTCAG | GTCACTGAATGAATGTGTGA |
| leg1a-5 | CATTCAGACAGCATATGCA | CATGAAACAGTGGTGTAGCA |
| leg1a-6 | GTTTGTGTGTGAAGTTTCAG | CACAACACGAGGTCAAATT |
| leg1a-7 | TTGCGTTTAATGTTCAACCGGC | CCCGTTTACACCAGTGCGTTT |
| leg1a-8 | ACACTCTCACCGTGGTAA | GGTGTAGTATATCCCCTTTA |
| leg1a-9 | GGTGAGCTTTCAGCAGAGCAGC | GCTGTTCATCCAGCTGAGCATG |
| leg1a-10 | TCATAAACTGGCAACTTCTG | TTAGACCCAGAATGCTGT |
| leg1a-11 | TGTTTTATCCAAATGCTGGC | CACCCTTAACAAACTGGAAT |
| leg1b-1 | CAGTGCCATTGTGGTCCAGTGG | CGGCCCACCTCAGTTCATGA |
| leg1b-2 | GGAAGCATCAGCCAATCA | ACGAATGCCCCATTCACA |
| leg1b-3 | TGTTAAGCATTTGCCCAATCGG | AAGAAAATCTCCCCCACACCCA |

**^a^**the HRE module of *leg1* upstream sequence in zebrafish

**^b^**the forward primer sequence orientation is 5’ end to 3’ end

**^c^**the reversed primer sequence orientation is 5’ end to 3’ end
