# Supplementary material for: Hypoxia-Inducible Factor 2 Alpha Is Essential for Hepatic Outgrowth and Functions via the Regulation of leg1 Transcription in the Zebrafish Embryo
Source: PLoS One. 2014 Jul 7;9(7):e101980. doi: 10.1371/journal.pone.0101980 (PMC4084947; doi:10.1371/journal.pone.0101980)
Supplement: Table S3 — The summary of HRE clusters in the upstream sequences of leg1 comparing with ChIP-PCR assay. (DOC) [file pone.0101980.s007.doc]

Table S3. The summary of HRE clusters in the upstream sequences of *leg1* comparing with ChIP-PCR assay

| **Gene name^a^** | **The cluster distance from transcription site^b^**  **(HREs >= 3)** | **The HRE module of *leg1*^c^** |
| --- | --- | --- |
| *leg1a* | -5393 ~ -5157 | **leg1a-7**, leg1a-6 |
| *leg1a* | -4515 ~ -4330 | **leg1a-5^*^** |
| *leg1a* | -3441 ~ -2966 | **leg1a-3, leg1a-4** |
| *leg1b* | -7178 ~ -6817 | none |
| *leg1b* | -6586 ~ -6241 | none |

^a^the gene symbol

^b^the location of *leg1a* or *leg1b* upstream sequences contain no less than three HREs in window of 500 bps in zebrafish

^c^the location of *leg1a* or *leg1b* upstream sequences responding to the HRE module of leg1

*Bold letters mean the positive results in ChIP-PCR assay
